# Natural polysaccharides as immunometabolic modulators in metabolic diseases: mechanisms and translational challenges

**DOI:** 10.3389/fimmu.2026.1903979

**Published:** 2026-07-23

**Authors:** Qiqi Wang, Lian Zhu, Fangfang He, Tongwei Zhu, Yuqing Tang, Chanjuan Chen, XinYa Zhang, Lei Wang

**Affiliations:** 1 Dongguan Hospital of Traditional Chinese Medicine, Guangzhou University of Chinese Medicine, Dongguan, China; 2 School of Pharmaceutical Science, Hunan University of Medicine, Huaihua, China; 3 College of Pharmacy, Jinan University, Guangzhou, China; 4 Chinese Medicine Guangdong Laboratory, Guangdong Hengqin, China

**Keywords:** gut microbiota, immunoregulation, macrophage polarization, metabolic diseases, metaflammation, natural polysaccharides

## Abstract

Metabolic disorders, especially obesity, type 2 diabetes mellitus, and metabolic dysfunction-associated steatotic liver disease, are becoming increasingly prevalent and have imposed a growing burden on public health systems. These diseases are commonly associated with insulin resistance and abnormal lipid metabolism, and increasing evidence indicates that immune imbalance and chronic low-grade inflammation are involved in their development. Natural polysaccharides are important bioactive components derived from plants, fungi, algae, and other natural sources. Current evidence supporting their beneficial effects in metabolic diseases is predominantly preclinical, mainly from cell-based and animal studies, while clinical evidence remains limited and heterogeneous. Natural polysaccharides have attracted interest as candidate bioactive compounds because some preparations have shown immunomodulatory and metabolic regulatory activities in experimental models. Their activities are closely related to structural features, including monosaccharide composition, glycosidic linkage types, molecular weight, branching structure, and chemical modification. Current preclinical evidence suggests that natural polysaccharides may alleviate metabolic inflammation by regulating macrophage polarization, suppressing pro-inflammatory cytokine production, modulating MAPK, NF-κB, AMPK, and related signaling pathways, and reshaping the gut microbiota-immune axis. These compounds may help improve several pathological features of metabolic disorders, such as insulin resistance, abnormal lipid metabolism, inflammatory injury, and tissue dysfunction. However, several challenges still limit their translation, including unclear structure-activity relationships, inconsistent preparation standards, limited bioavailability, and insufficient well-designed clinical trials. Therefore, this review provides an overview of the natural sources, structural properties, immunomodulatory actions, and therapeutic prospects of natural polysaccharides in metabolic diseases, with a focus on their involvement in immune regulation and metabolic inflammation.

## 1 Introduction

In recent years, obesity, metabolic dysfunction-associated steatotic liver disease (MASLD), and type 2 diabetes mellitus (T2DM) are common metabolic disorders that have imposed an increasing burden on global health (1). These conditions are highly interconnected and share common pathological features such as insulin resistance, lipid metabolism disorders, and chronic low-grade inflammation (2–4). Disrupted crosstalk between immune responses and metabolic regulation is increasingly recognized as a fundamental mechanism contributing to the development and progression of metabolic diseases (5). Nutrient overload, obesity, and metabolic stress can reshape local immune niches across key metabolic organs, such as liver, adipose tissue, intestine, and vasculature. This remodeling is characterized by aberrant macrophage activation and polarization imbalance, dysregulation of T-cell subsets, sustained release of pro-inflammatory cytokines, excessive activation of inflammatory signaling pathways, and impaired resolution of inflammation (6, 7). These immune changes can further affect insulin signal transduction, increase lipid deposition, worsen mitochondrial dysfunction, and lead to more serious tissue injury. As these changes continue, metabolic disorder and immune inflammation may influence each other and form a repeated harmful cycle. Therefore, metabolic diseases should not only be understood as disorders of glucose and lipid metabolism. They are also complex immunometabolic diseases, in which metabolic stress and immune imbalance are closely connected (5, 8). Although several drugs have been used for metabolic diseases, their clinical use still has some limitations, including adverse effects, different responses among patients, and the difficulty of acting on the complicated immunometabolic networks involved in disease progression.

With the increasing prevalence of metabolic diseases, researchers have paid more attention to natural bioactive compounds with potential metabolic and immunomodulatory activities; however, their efficacy and safety require preparation-specific evaluation (9, 10). Natural polysaccharides are widely found in algae, fungi, plants, and some other natural materials, and many of them are considered bioactive polysaccharides. They are also important components in dietary fibers and medicinal natural products. Common examples include polysaccharides from *Auricularia auricula, Lycium barbarum, Ganoderma lucidum*, and *Astragalus* (11). These polysaccharides are usually made up of different monosaccharides, and these monosaccharides are connected by glycosidic bonds. In general, they show relatively low toxicity, good biocompatibility, and can regulate several biological targets at the same time (12). The structural complexity of polysaccharides is affected by many factors, including monosaccharide composition, molecular weight, glycosidic bond types, branching form, three-dimensional structure, and chemical modification. These differences also influence their biological functions, and different structures may lead to different activities (13).

Notably, many natural polysaccharides exhibit considerable immunomodulatory potential. Depending on their structural motifs and spatial conformations, some polysaccharides may interact with pattern recognition receptors expressed on macrophages, dendritic cells, intestinal immune cells, and other immune cell populations, such as Toll-like receptors, mannose receptors, Dectin-1, and complement receptors (14–16). These interactions may regulate immune cell activation, macrophage polarization, antigen presentation, cytokine secretion, and inflammatory homeostasis. The downstream mechanisms involve multiple immune- and inflammation-related signaling pathways, such as PI3K/Akt, MAPK, NF-κB, and inflammasome-related pathways (17, 18). Therefore, natural polysaccharides should not be regarded merely as metabolic regulators; rather, they represent promising natural immunomodulatory agents that may support the development of novel approaches for metabolic disease prevention and therapy.

Emerging evidence suggests that natural polysaccharides improve metabolic disorders not only by directly regulating metabolic processes, but may also be accompanied by coordinated changes in the immune-microbiota-metabolism network (19, 20). On the one hand, natural polysaccharides can regulate intracellular signaling pathways closely associated with inflammation and metabolic homeostasis. For example, some natural polysaccharides can stimulate AMPK-dependent signaling, thereby enhancing glucose utilization, fatty acid catabolism, and mitochondrial activity. Beyond these metabolic effects, AMPK activation may also suppress metabolic inflammation by limiting NF-κB overactivation, oxidative stress, and pro-inflammatory cytokine production, while promoting macrophage transition toward anti-inflammatory or tissue-repair phenotypes (21, 22). In addition to AMPK, natural polysaccharides can modulate PI3K/Akt, MAPK, and PPAR-related signaling networks, which may improve insulin sensitivity and lipid handling while reshaping immune responses within metabolically inflamed tissues (23, 24). Due to their poor digestibility by host enzymes, numerous natural polysaccharides can pass into the colon and participate in microbial fermentation. Through this process, they may be associated with altered gut microbial communities and increased production of microbial metabolites such as short-chain fatty acids (25, 26). Beyond their role in energy metabolism, short-chain fatty acids act as key microbial signals that help maintain gut barrier integrity, decrease lipopolysaccharide leakage, shape Treg and macrophage responses, and reduce chronic systemic inflammation (27). In addition, polysaccharide intervention has been associated with changes in bile acid metabolism and gut microbiota composition, which may be linked to altered FXR- and TGR5-related signaling, lipid handling, and energy balance (28, 29). Collectively, natural polysaccharides may intervene in obesity, T2DM, MASLD/MASH, and atherosclerosis by coordinately regulating inflammation, the gut microbiota, and metabolic signaling networks. In this process, immunomodulation may represent a potential link between microbial alterations and metabolic improvement, although causal relationships require further experimental validation.

However, although current findings highlight the immunometabolic benefits of natural polysaccharides, several key obstacles continue to limit their further development for metabolic disease management. At present, the relationship between polysaccharide structure and biological activity is still not clear enough, and it is also difficult to tell how much of their effect comes from direct immune regulation and how much comes from gut microbiota-related actions. In addition, polysaccharides used in different studies may come from different sources, and the extraction methods, purification processes, structural analysis methods, doses, and experimental models are not always the same; these differences make the results harder to compare and repeat. Their absorption, degradation, fermentation, transformation, and metabolic fate in the body also need to be further studied, and reliable clinical evidence is still insufficient. Beyond these biomedical and translational challenges, the sustainable sourcing and production of natural polysaccharides also deserves attention. Recent studies on food-system sustainability, ecosystem-based natural resource management, and circular utilization of agricultural biomass further support the development of natural polysaccharides as renewable bioresources for future food and health applications. Therefore, this review summarizes the immunological basis of metabolic diseases and discusses recent progress in natural polysaccharides as immune-regulating agents.

## 2 Immune microenvironment dysregulation in metabolic diseases

**Figure 1:**
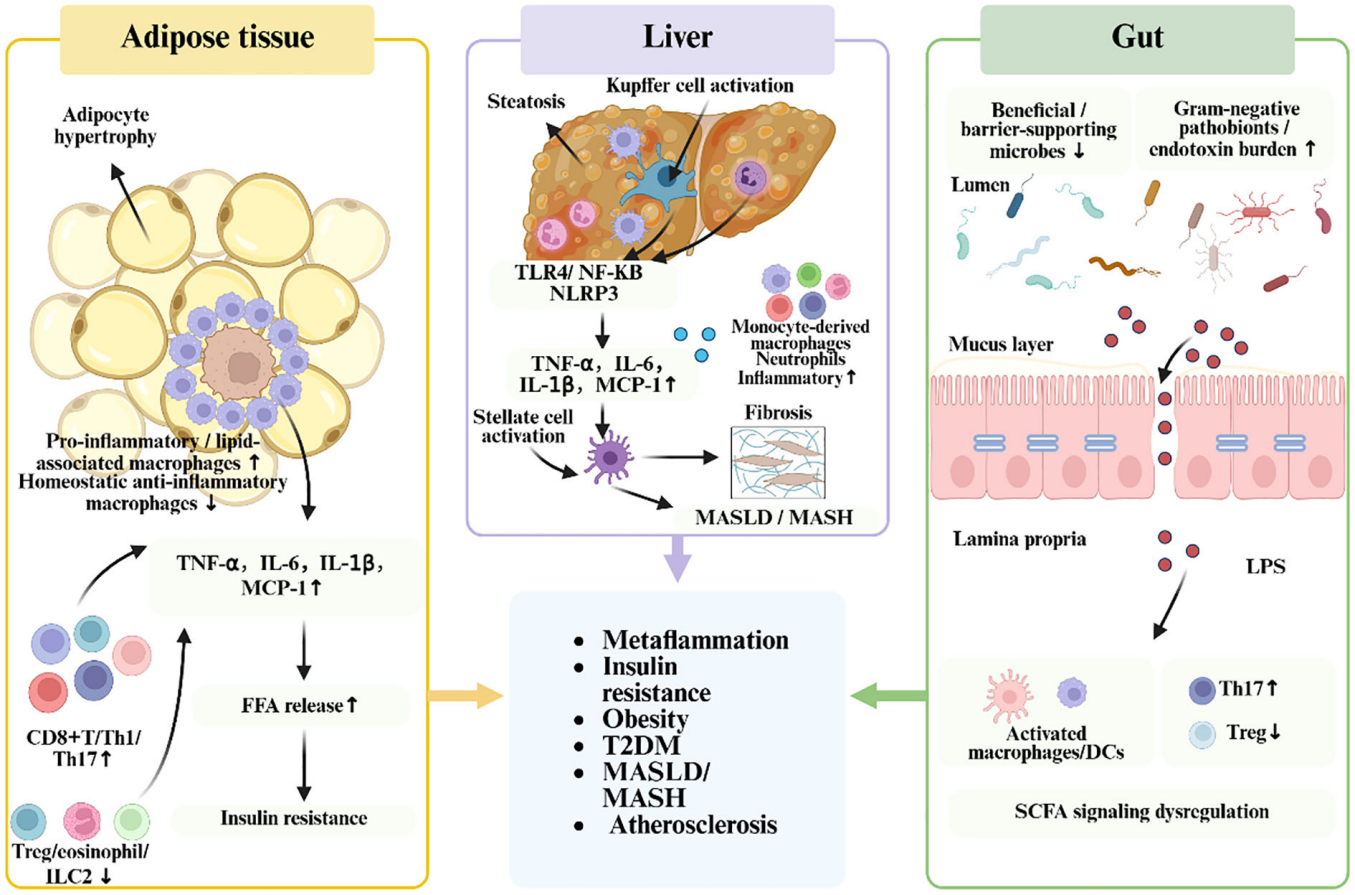
Immune dysregulation in metabolic diseases.

In metabolic diseases, immune homeostasis is often disturbed in several important organs related to metabolism, and adipose tissue, the liver, and the intestine are usually the main affected sites. In adipose tissue, enlarged adipocytes and altered macrophage phenotypes drive persistent inflammation and impair insulin sensitivity. In the liver, activation of Kupffer cells, secretion of inflammatory cytokines, and inflammasome-related signaling promote hepatic steatosis, MASH development, and fibrotic remodeling. In the gut, microbial dysbiosis, impaired barrier integrity, LPS translocation, and Th17/Treg imbalance further amplify systemic metabolic inflammation. These interconnected immune alterations form a self-reinforcing network that drives metaflammation and metabolic dysfunction (**Figure 1**).

### 2.1 Phenotypic heterogeneity and polarization of adipose tissue macrophages

Adipose tissue macrophages (ATMs) are central immune components of adipose tissue and play important roles in maintaining local inflammatory balance, lipid handling, tissue remodeling, and insulin sensitivity under physiological conditions (30, 31). In healthy adipose tissue, ATMs are enriched in M2-like phenotypes that use OXPHOS and FAO as primary metabolic pathways and produce anti-inflammatory cytokines such as IL-10 and TGF-β (32). These cells generally rely more on oxidative phosphorylation (OXPHOS) and fatty acid oxidation (FAO), whereas pro-inflammatory M1-like macrophages are usually associated with enhanced glycolytic metabolism (32–35). However, this M1/M2 metabolic distinction should be viewed as a simplified framework rather than a fixed rule, because ATM function is strongly shaped by local nutrient availability, adipocyte-derived signals, and tissue stress.

During obesity, adipocyte hypertrophy, local hypoxia, mechanical stress, and adipocyte death reshape the adipose immune microenvironment. Enlarged or stressed adipocytes release chemokines and inflammatory mediators, including CCL2, IL-6, and TNF-α, which promote monocyte recruitment and macrophage accumulation within adipose tissue (36–38). Recruited macrophages often localize around dying adipocytes to form crown-like structures (CLS). These structures participate in lipid clearance and tissue remodeling, but they also represent inflammatory niches that contribute to persistent adipose tissue inflammation. In this context, metabolic stress signals such as excessive free fatty acids, endotoxin exposure, and oxidative stress can promote inflammatory activation of ATMs and reduce their tissue-repair functions, thereby linking adipose tissue expansion to systemic metabolic dysfunction (39, 40).

Functionally, obesity-associated ATMs often show increased expression of inflammatory markers such as CD11c and iNOS and produce higher levels of IL-1β, TNF-α, and IL-6 (41, 42). These cytokines can impair insulin action in adipocytes and other metabolic tissues. For example, TNF-α promotes inhibitory serine phosphorylation of IRS-1, thereby weakening insulin receptor downstream signaling, while sustained IL-6 exposure can induce SOCS3 expression and further interfere with insulin signal transduction. As a result, ATM-derived inflammatory mediators contribute to insulin resistance, enhanced lipolysis, increased free fatty acid release, and metabolic stress in distal organs, particularly the liver.

However, the traditional M1/M2 classification cannot fully explain the complex changes in ATM phenotypes (43, 44). Recent single-cell studies have shown that ATMs do not represent a single homogeneous population but comprise multiple subsets different macrophage subsets, such as metabolically activated macrophages (MMe) and Trem2^+^ lipid-associated macrophages (LAMs) (45). These macrophage subsets are involved in lipid handling, inflammatory regulation, and tissue adaptation. Overall, ATM infiltration, crown-like structure formation, inflammatory cytokine release, and macrophage metabolic reprogramming constitute key adipose tissue-specific mechanisms linking obesity to chronic low-grade inflammation and insulin resistance (46).

### 2.2 Dysregulation of T cells and other immune cell subsets

In obesity, T2DM, and MASLD, chronic low-grade inflammation is not driven only by macrophages, but also involves coordinated changes in adaptive and innate immune-cell populations (47–49). These immune alterations occur in a tissue-specific manner. In adipose tissue, altered T-cell composition contributes to macrophage recruitment and inflammatory amplification. In the liver, lymphocytes and innate immune cells interact with Kupffer cells, hepatocytes, and hepatic stellate cells during the progression from steatosis to steatohepatitis and fibrosis. In the intestine, dysregulated mucosal immunity can further aggravate barrier dysfunction and systemic metabolic inflammation.

Among adaptive immune cells, imbalance of CD4^+^ T cell subsets is particularly important (50). Under physiological conditions, regulatory T cells (Tregs) help maintain immune tolerance and limit excessive inflammatory activation through mediators such as IL-10 and TGF-β (51, 52). During metabolic stress, Treg abundance or function is often reduced, whereas pro-inflammatory Th1 and Th17 responses are increased. This shift promotes the production of IFN-γ, TNF-α, and IL-17, which can activate macrophages, amplify adipose tissue inflammation, and interfere with insulin signal transduction (53). Therefore, Th1/Treg and Th17/Treg imbalance represents an important link between immune dysregulation and metabolic dysfunction.

The balance between Th17 cells and Tregs is strongly influenced by immune-cell metabolic programming. Activated effector T cells usually use more glucose, and they often depend more on aerobic glycolysis. This metabolic change can support cell proliferation and the production of inflammatory cytokines. Th17 cells are a type of effector CD4^+^ T cells, and they also show stronger glycolytic metabolism, with increased activity of the pentose phosphate pathway. In contrast, Tregs usually depend more on FAO and OXPHOS, and this helps them maintain their immune-suppressive function (54). When glucose availability is reduced, Th17 differentiation may be inhibited, while Treg differentiation and function may be promoted, which suggests that nutrient and metabolic conditions can directly affect the balance of T cell subsets. Lipid metabolism also contributes to T cell fate determination. Molecules such as mTOR, SREBP, PPARγ, and ACC1 regulate fatty acid uptake, lipid synthesis, and T cell differentiation (55–57). Increased lipid synthesis is often associated with effector T cell and Th17 responses, whereas Tregs preferentially use exogenous fatty acids and depend on FAO to support their function.

Beyond T cells, several other immune-cell populations are also involved in amplifying inflammatory responses during metabolic disease progression. B cells have received increasing attention in MASLD. The liver has prominent immunological functions, and B cells constitute one of the major lymphocyte populations present in hepatic tissue (58). During MASLD progression, B-cell activation in the liver is accompanied by enhanced inflammatory gene signatures and elevated production of IL-6 and TNF-α, thereby contributing to chronic hepatic inflammation and fibrosis (59). Whether hepatic B-cell accumulation actively drives MASH or occurs secondary to disease progression remains unresolved; nevertheless, available evidence indicates that B cells are involved in the worsening of MASLD. Beyond adaptive immune responses, innate immune components, including neutrophils, dendritic cells, and NK cells, are also implicated in the inflammatory remodeling associated with metabolic diseases. For example, neutrophils mainly rely on glycolysis for energy and can release reactive oxygen species and chemokines in inflammatory environments (60). Changes in the metabolism of dendritic cells can affect their antigen presentation and T cell activation (61), while the function of NK cells is related to glycolysis, FAO, and lipid accumulation (62). These immune cells also take part in the changes of the immune microenvironment during metabolic diseases.

Overall, decreased Treg function, increased Th1/Th17 cells, and abnormal activation of B cells, neutrophils, dendritic cells, and NK cells all contribute to the chronic inflammatory microenvironment in metabolic diseases. The main process is not only immune activation; it also involves the interaction between metabolic changes in immune cells and inflammatory signaling, and these two processes can further strengthen each other. The Th17/Treg axis, together with related metabolic pathways such as glycolysis, FAO, OXPHOS, and mTOR signaling, may serve as important links between immune imbalance and metabolic dysfunction.

### 2.3 Chronic inflammatory cytokine networks and metabolic interactions

In metabolic diseases, chronic low-grade inflammation represents a key link between immune dysregulation and metabolic disturbance (63). Rather than being restricted to a single tissue or immune-cell population, metabolic inflammation is sustained by a cytokine network that connects adipose tissue, liver, intestine, pancreatic islets, and the systemic circulation. Immune cells and parenchymal cells within these tissues continuously exchange inflammatory and metabolic signals, thereby forming a self-amplifying cycle between immune activation and metabolic dysfunction (64).

Among the inflammatory mediators involved in metabolic diseases, TNF-α, IL-6, and IL-1β are particularly important (35). In adipose tissue, TNF-α and IL-6 derived from macrophages and stressed adipocytes can impair insulin responsiveness, enhance lipolysis, and increase free fatty acid release. These changes not only aggravate local adipose inflammation but also increase lipid flux to the liver. In pancreatic islets, sustained exposure to inflammatory cytokines, especially IL-1β, can impair β-cell function and contribute to defective insulin secretion. Therefore, cytokine-mediated crosstalk provides an important mechanism through which local immune activation is translated into systemic glucose and lipid metabolic abnormalities.

In MASLD/MASH, inflammatory responses and hepatic lipid metabolic disorders reinforce one another. Excess free fatty acids, cholesterol accumulation, and gut-derived endotoxin can activate Kupffer cells and recruited monocyte-derived macrophages, leading to amplification of the hepatic inflammatory microenvironment (65). Inflammatory mediators produced in the liver further promote hepatocyte injury, metabolic stress, and fibrogenic responses. In addition to macrophages, B cells, T cells, neutrophils, and hepatic parenchymal cells may also participate in shaping the inflammatory landscape of metabolic liver disease, indicating that MASLD/MASH progression is driven by coordinated interactions among multiple hepatic and extrahepatic cell types.

Gut-derived inflammatory signals further intensify the inflammation–metabolism cycle. Under conditions such as obesity and high-fat feeding, impaired intestinal barrier integrity facilitates the translocation of microbial products into the portal and systemic circulation, thereby contributing to metabolic endotoxemia (66, 67). These gut-derived signals can enhance inflammatory responses in adipose tissue and liver, while persistent systemic inflammation may further weaken intestinal barrier function. As a result, gut microbial imbalance, barrier disruption, immune activation, and metabolic dysfunction can mutually reinforce each other.

In summary, inflammation in metabolic diseases is not a separate event, but a process involving several related changes, including metabolic changes in immune cells, release of inflammatory factors, gut microbiota dysbiosis, lipid accumulation, and impaired insulin signaling. Therefore, regulating immune-cell metabolism, excessive inflammatory pathway activation, intestinal barrier injury, and gut microbial imbalance may be useful for the management of metabolic diseases. In this situation, natural polysaccharides have received attention, because they may act on several immunometabolic targets at the same time.

## 3 Sources and structural characteristics of immunomodulatory polysaccharides

**Figure 2:**
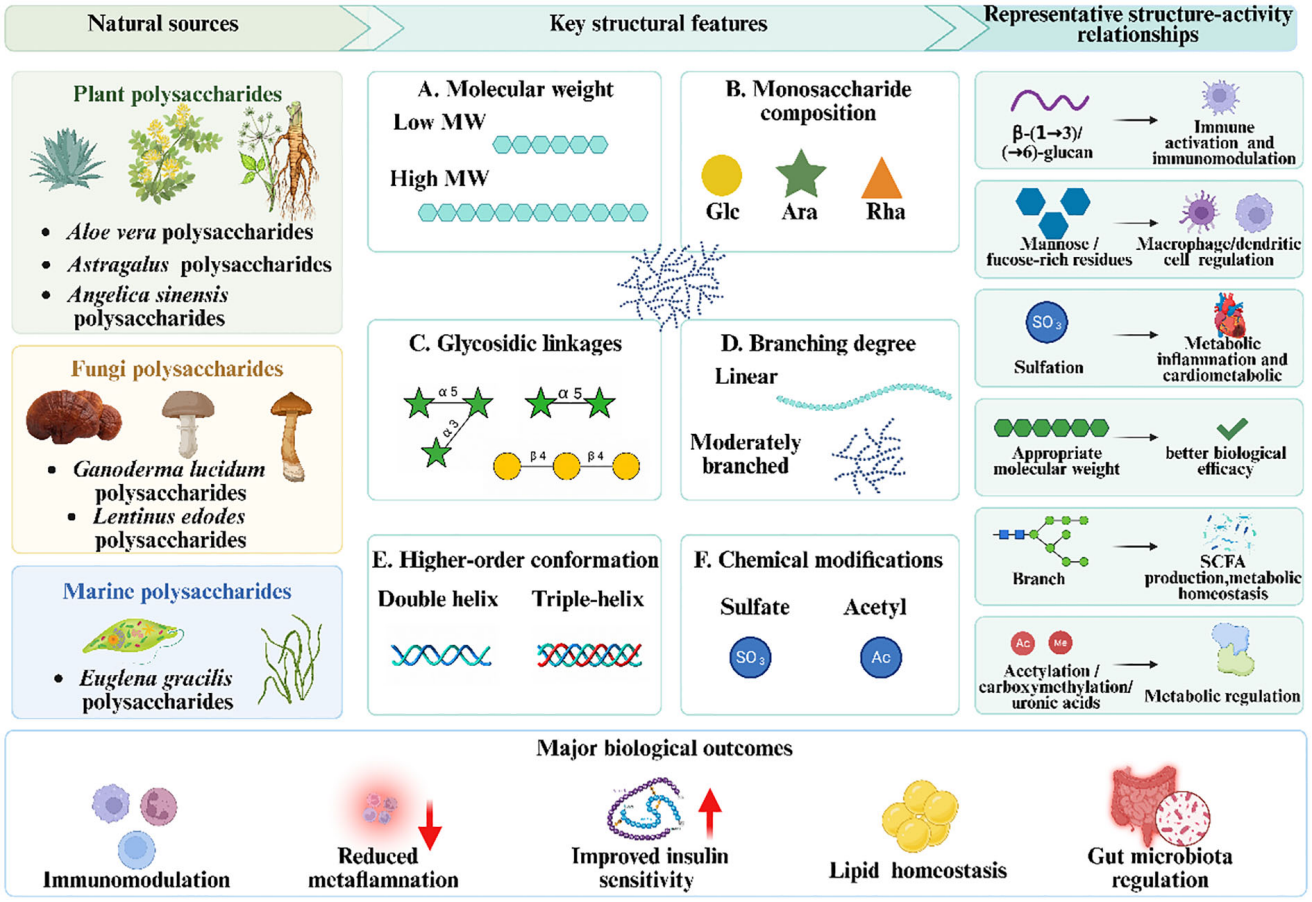
Sources and structure-activity relationships of natural polysaccharides.

Natural polysaccharides mainly come from plants, fungi, and marine organisms, and many of them have certain immune-regulating potential. The functions of these macromolecules are closely related to their structures, such as molecular weight, sugar composition, linkage types, branching patterns, spatial structure, and modified groups. These structural features can influence receptor recognition, gut microbial fermentation, immune-cell regulation, and metabolic effects, so they also affect the immunomodulatory role of polysaccharides in metabolic diseases (**Figure 2**).

### 3.1 Sources classification

Plants, fungi, and marine organisms are the main sources of natural polysaccharides that have immune-regulating activity (68). Polysaccharides from different sources are often different in monosaccharide composition, glycosidic linkage types, chemical modification, and branching structure, and these structural differences can affect their immune-regulating properties and their possible therapeutic effects in metabolic diseases (69). Increasing evidence suggests that these polysaccharides, beyond serving as dietary bioactive components, can regulate immune homeostasis and metabolic balance, thereby contributing to the prevention and treatment of obesity, type 2 diabetes, and non-alcoholic fatty liver disease.

Plant polysaccharides (PPS) are natural biopolymers widely distributed in plants. They mainly include starch, cellulose, and pectin (70). From a sustainability perspective, the source and production pathway of polysaccharides are important for their future translation. Polysaccharide-rich biomass from plants, fungi, algae, and agro-industrial by-products may provide renewable materials for functional foods, nutraceuticals, and biomedical applications. For example, date palm fruits, seeds, and other by-products represent underutilized resources that can be valorized within a circular bioeconomy framework (71–73). Among them, cellulose is an important cell-wall polysaccharide, whereas starch mainly functions as a storage polysaccharide in plant organs. Based on their plant sources, PPS can be obtained from stems, leaves, flowers, fruits, and roots (74). PPS Plant polysaccharides usually consist of diverse monosaccharide units connected through glycosidic linkages, and their molecular weights may vary from tens of thousands to several million daltons (75). Their common monosaccharide components include galactose, fructose, glucose, arabinose, xylose, rhamnose, fucose, mannose, and uronic acid (76). The composition varies among different plants; for example, acemannan from *Aloe vera* is mainly composed of acetylated mannose, galactose, and glucose (77), while *Angelica sinensis* polysaccharides mainly contain glucose, glucuronic acid, galactose, and arabinose (78). Structurally, PPS are commonly connected by α-(1→6)-D, α-(1→4)-D, and β-(1→4)-D glycosidic bonds (79). The bioactivities of PPS are strongly influenced by their monosaccharide composition, linkage patterns, molecular size, and functional group characteristics (70). The safety of some herb-derived polysaccharides has been evaluated in preclinical studies through acute toxicity tests, repeated administration toxicity tests, developmental and reproductive toxicity tests, and genetic toxicity tests (80). They exhibit various functional activities, including immunomodulatory, antioxidant, antitumor, antidiabetic, hepatoprotective, antihypertensive, and antidepressant effects (70, 81, 82). Therefore, PPS have attracted increasing attention as important natural bioactive components because of their wide distribution, structural diversity, and multiple biological activities.

*β*-glucans are the major representatives of fungal polysaccharides, which are abundant in medicinal fungi such as *Cordyceps*, and *Lentinus edodes*, *Ganoderma lucidum* (83). These polysaccharides typically consist of a *β*-(1→3)-linked backbone with *β*-(1→6) branches, and some exhibit a characteristic triple-helix conformation, which is considered essential for their biological activity. Compared with plant polysaccharides, fungal polysaccharides often have more direct immune-regulating effects through receptor recognition (84). Some receptors, such as Dectin-1, Toll-like receptors, and other related pattern-recognition receptors, can recognize fungal polysaccharides. After this recognition, fungal polysaccharides may regulate the activation of macrophages, dendritic cells, and T cells, and then help maintain immune balance and control inflammation. Recent studies also show that fungal polysaccharides may improve metabolic disorders by changing the gut microbial community, protecting the intestinal barrier, and regulating lipid metabolism (85). Consistent with these preclinical findings, a recent randomized placebo-controlled exploratory study in patients with T2DM showed that yeast β-1,3/1,6-glucan supplementation lowered insulin resistance after 8 weeks and reduced TNF-α after 4 weeks, suggesting the translational relevance of fungal β-glucans in metabolic inflammation (86).

Marine polysaccharides mainly come from brown algae, red algae, and green algae, and fucoidan and alginate are usually considered representative compounds among them (87). These polysaccharides usually have many sulfate groups, and these groups give them a strong negative charge and some special structural features, which may support their biological activities (88). Marine polysaccharides have several biological activities, including anti-inflammatory, antioxidant, and immune-regulating effects. Because of these properties, some representative compounds, such as agar and alginate, have been widely used in biomedical and therapeutic fields, especially in wound repair, drug delivery, and tissue engineering (89). With the increasing incidence of hyperlipidemia, obesity, and diabetes, low-cost therapeutic strategies are urgently needed to slow the progression of atherosclerosis effectively and economically (90, 91). Marine polysaccharides have a long history of human consumption and are abundant and inexpensive. Among them, ulvan, laminarin sulfate, and fucoidan demonstrated beneficial effects in animal models for alleviating atherosclerosis and related risk factors (92).However, human evidence for marine polysaccharides remains inconsistent, as a randomized double-blind placebo-controlled trial in obese nondiabetic adults reported that fucoidan supplementation for 90 days did not markedly improve insulin resistance or other cardiometabolic markers, highlighting the gap between preclinical efficacy and clinical translation (93).

Overall, the structural diversity of natural polysaccharides from different sources determines their functional specificity in immune recognition, signaling pathways, and metabolic regulation. These differences influence their interactions with pattern recognition receptors and ultimately affect their therapeutic efficacy in metabolic diseases. Therefore, a systematic classification based on sources provides an important foundation for understanding their structure–activity relationships and immunoregulatory mechanisms. From a sustainability perspective, the source and production pathway of polysaccharides are important for their future translation. Polysaccharide-rich biomass from plants, fungi, algae, and agro-industrial by-products may provide renewable materials for functional foods, nutraceuticals, and biomedical applications. For example, date palm fruits, seeds, and other by-products represent underutilized resources that can be valorized within a circular bioeconomy framework.

### 3.2 Structure-activity relationship

Natural polysaccharides are important biological macromolecules with diverse functional properties, such as antidiabetic, anti-inflammatory, gut microbiota-modulating, antioxidant, and immune-regulating activities (94). The functional properties of polysaccharides depend strongly on specific structural parameters, including molecular size, monosaccharide composition, linkage patterns, branching, and conformational features. This connection between molecular structure and biological effect is generally defined as the structure-activity relationship (SAR). Polysaccharides usually exist as structurally heterogeneous macromolecules rather than as molecules with a uniform and definite architecture. Their physicochemical and biological properties are shaped by multiple structural parameters, such as molecular size, monosaccharide profile, glycosidic bond pattern, branching extent, three-dimensional conformation, and chemical substituents. These features jointly influence their solubility, viscosity, stability, bioavailability, and receptor-binding capacity (14, 95). Therefore, their roles in regulating energy metabolism, immune responses, and gut microecology are usually not determined by one structural parameter alone, but by the combined effects of multiple structural features. Accumulating evidence suggests that natural polysaccharides contribute to energy homeostasis through multiple structure-dependent pathways, including the regulation of cellular metabolism, remodeling of the gut microbiota-metabolic axis, and modulation of immunometabolic interactions. Therefore, their biological effects are closely linked to their molecular characteristics. **Table 1** summarizes their main sources, representative structural characteristics, and major targeted metabolic diseases.

**Table 1:** Sources, structural characteristics and major targeted metabolic diseases of common polysaccharides.

Polysaccharides	Sources	Molecular weight (Da)	Monosaccharides	Effect	Mechanism	Disease	Targeted pathway	Therapeutic outcomes
*Angelica sinensis polysaccharides*	Rhizome	5.1×10^3^-2.3× 10^6^	Glc, Man, Gal, Rha, Ara, Xyl	Inhibiting TNF-α, IL-1β, and TNF-α expression, inhibiting SOD and CAT activity, decreasing MDA content, inhibiting caspase-3 and Bax/Bcl-2 expression	Activating the BDNF/TrkB/CREB signaling pathway	T2DM (96)	PI3K/AKT	promotes hematopoiesis and thrombopoiesis
*Pumpkin (Cucurbita moschata) polysaccharides*	Fruit	Not reported	Gal, Glc, Ara, Xyl, GlaA	Educating TG, TC, and plasma LDL-C, increasing the levels of fecal fat, cholesterol, and plasma HDL-C	Increasing the binding capacity of fat and cholesterol	Obesity (97)	Not reported	demonstrated the hypolipidemic activity
*Lycium barbarum polysaccharides*	Rhizome	1× 10^4^-2.3× 10^6^	Rha, Fuc, Ara, Gal	Educating TG, TC and LDL-C levels, increasing HDL-C and SCFA	Improving IR and fatty acid oxidation, activating the adenosine monophosphate activated protein kinase CoA carboxylase pathway	Obesity (98)	the activation of nuclear factor kappa B and the inhibition of the nucleotide-binding and oligomerization domain-like receptor protein 3/6 inflammasome pathway	improve dyslipidemia, promotes energy expenditure, reduces body weight, and alleviates non-alcoholic steatohepatitis
*Schisandra Caulis Caulis polysaccharides*	Fruit	Not reported	Rha, Fuc, Ara, Xyl, Man, Glc, Gal	Reducing AST, ALT, TG, TC, and LDL-C, increasing HDL-C	Regulating UGP2, UGDH, ACC and FAS expression	NASH (99)	Regulate the metabolism of pyruvate and nicotinic acid.	facilitate the recovery of the liver morphology, structure, and function.
*Poria cocos polysaccharides*	Rhizome	4.1× 10^4^-5× 10^5^	Glc, Fuc, Ara, Xyl, Man, Gal	Reduced serum TNF-α, IL-6, NO, LDL-C, TG and TC levels, decreasing MDA, and increasing SOD	Inhibiting the TLR4/NF-κB pathway	AS (100)	TLR4/NF-κB p65 signaling pathway	improved the pathological characteristics of AS.
*Poria cocos polysaccharides*	Rhizome	4.1× 10^4^-5× 10^5^	Glc, Fuc, Ara, Xyl, Man, Gal	Increasing lipid utilization, decreasing lipid synthesis and absorption	Regulating fatty acid metabolism, bile acid metabolism, and tricarboxylic acid cycle	NAFLD (101)	metabolic pathway of the primary BA biosynthesis and fatty acid biosynthesis	promote gut motility and glucose metabolism
*Walnut green husk polysaccharides*	Walnut green husk	4.813 ×10^6^	GlcA, Ara, Gal	Regulating glucose and lipid metabolism	reducing liver MDA levels and enhancing GSH-Px and T-SOD activity	NAFLD (102)	regulate glycolytic pathway	regulate body weight, reduce glucose intolerance
*Jackfruit pulp polysaccharides*	Jackfruit pulp	1.668× 10^3^	Ara, Gal, Glc, GalA	Reduce lipid accumulation and modify lipid metabolism	activated the HSL/LPL-CPT1 pathway connected with lipolysis and the PPARα-CPT1 pathway related to β-oxidation of fatty acids.	NAFLD (103)	activate PPAR and AMPK signaling pathways.	ameliorate inflammation and liver injury
Dioscorea opposita polysaccharides	Rhizome	4× 10^3^-7× 10^5^	Glu, xyl, gala, Ara, ManFuc	Reducing blood sugar	Not reported	T2DM (104)	Not reported	hypoglycemic effect

Among the structural characteristics of polysaccharides, molecular weight plays an important role in determining their physicochemical properties and biological activities (105). However, the influence of molecular weight on polysaccharide bioactivity varies depending on the source, structural context, and biological model. Polysaccharides from different sources may have an optimal molecular weight range in which stronger immunomodulatory or metabolic regulatory activities are observed (106). From the perspective of immune regulation, the difference in molecular weight also affects the way polysaccharides interact and bind with the surface receptors of immune cells. Low molecular weight polysaccharides are generally more easily diffused and may have a faster contact with macrophages, dendritic cells, or the surface receptors of intestinal epithelial cells, thereby influencing cytokine secretion and inflammatory signal transduction (107). In contrast, high molecular weight polysaccharides may form a more stable spatial structure or aggregation state, which is conducive to multivalent binding with pattern recognition receptors, but they may also be limited in tissue penetration due to their larger molecular size (108). Therefore, different molecular weight ranges may lead to different effects of polysaccharides in regulating macrophage polarization, immune cell activation, and the release of inflammatory factors.

Monosaccharide composition is also an important structural basis for polysaccharide activity. The monosaccharide profile of polysaccharides, including both the species and relative ratios of sugars such as mannose, glucose, arabinose, galactose, rhamnose, fucose, and uronic acids, can affect the interactions of polysaccharides with pattern recognition receptors, glycoside hydrolases from gut microbiota, and host metabolic pathways (109). Different monosaccharide residues may also determine the preference of polysaccharides for immune recognition. For example, glucose-rich polysaccharides, especially certain β-glucans, are often associated with Dectin-1- or CR3-mediated immune recognition and can affect the activation of macrophages and dendritic cells. Mannose-containing polysaccharides may bind to mannose receptors on the surface of macrophages or dendritic cells, thereby influencing antigen presentation and cytokine secretion. Fucose-, rhamnose-, or uronic acid-containing polysaccharides may participate in immune homeostasis by affecting cell adhesion, inflammatory mediator release, or gut microbiota metabolism. In addition, monosaccharide composition and its relative proportions can also influence the utilization preference of gut microbiota for polysaccharides, thereby altering the production of microbial metabolites such as short-chain fatty acids and further affecting Treg cell differentiation, macrophage polarization, intestinal barrier function, and systemic low-grade inflammation.

The nature of glycosidic bonds and their linkage patterns represents a key structural basis for understanding the SAR of polysaccharides (110). Different *α*- or *β*-glycosidic linkages can affect chain flexibility, spatial conformation, and resistance to digestion. For example, some specific β-glucan structures can be recognized by receptors on immune cells, and this recognition may further affect macrophage activation, dendritic cell function, and other immune-cell responses (111). In contrast, some polysaccharides have glycosidic linkages that are not easily hydrolyzed by host digestive enzymes, and because of this resistance, they can enter the colon. In the colon, gut microbiota can use and ferment these polysaccharides. During microbial fermentation, polysaccharides may be changed into bioactive metabolites, such as short-chain fatty acids, and these metabolites can further regulate glucose and lipid metabolism, while also affecting inflammatory signaling. Besides linkage types, the degree of branching and higher-order conformation can also influence biological activity (112). Moderate branching may provide more potential binding sites and increase the contact opportunities between polysaccharides and immune receptors, such as TLR2/4, Dectin-1, mannose receptor, and CR3, thereby influencing NF-κB, MAPK, PI3K/Akt, and inflammasome-related signaling pathways. Through these pathways, polysaccharides can further regulate macrophage phenotypic transformation, inflammatory cytokine release, and the activation state of immune cells. However, a higher degree of branching does not necessarily lead to stronger activity. Excessive branching may mask some key binding regions or alter the original spatial conformation of polysaccharides, thereby reducing receptor recognition efficiency. Therefore, the influence of branching structure on immune effects depends on whether it can maintain a spatial conformation suitable for receptor recognition. A moderate degree of branching may provide more receptor-binding sites, and it may also increase the contact between polysaccharides and immune cells or microbial enzymes. Stable helical conformations or aggregated states may help keep molecular stability and support receptor recognition, but these effects are still related to the polysaccharide source, backbone structure, and overall conformation.

Chemical modifications, such as acetylation, carboxymethylation, and sulfation, can further change the charge distribution, water solubility, and spatial structure of polysaccharides, and these changes may also affect their biological functions (113). For example, sulfated polysaccharides usually carry strong negative charges, and these charges may help them interact more easily with protein receptors or cell membrane structures (114). Carboxymethylation and acetylation may improve the solubility of polysaccharides, or change their molecular flexibility. However, the effects caused by chemical modification are not only decided by how much modification is introduced. These effects are also closely related to the degree of substitution, the specific substitution sites, and the original structural framework of the polysaccharide.

Taken together, the SAR of natural polysaccharides should not be explained only from one structural factor, but should be considered together with structure, metabolism, gut microbiota, and immune regulation. Natural polysaccharides may influence immunometabolic homeostasis mainly through two related ways. On the one hand, some polysaccharides can act on intracellular signaling pathways, including AMPK, PPAR, PI3K/Akt, NF-κB, and MAPK; through these pathways, they may regulate glucose uptake, lipid metabolism, mitochondrial function, and inflammatory responses. On the other hand, the structural characteristics of polysaccharides can also affect whether they can be used by gut microbiota, and this process further influences the production of microbial metabolites, such as short-chain fatty acids and bile acid-related products. In this way, polysaccharides may further affect host energy metabolism and immune balance in an indirect manner. However, the causal relationship between polysaccharide structure and biological function is still not clear enough. Future studies should combine more detailed structural analysis, *in vivo* metabolic fate research, gut microbiota intervention, and receptor-recognition experiments. These studies may provide a more solid basis for the targeted development and application of natural polysaccharides in metabolic diseases.

## 4 Core immunoregulatory mechanisms of natural polysaccharides

**Figure 3:**
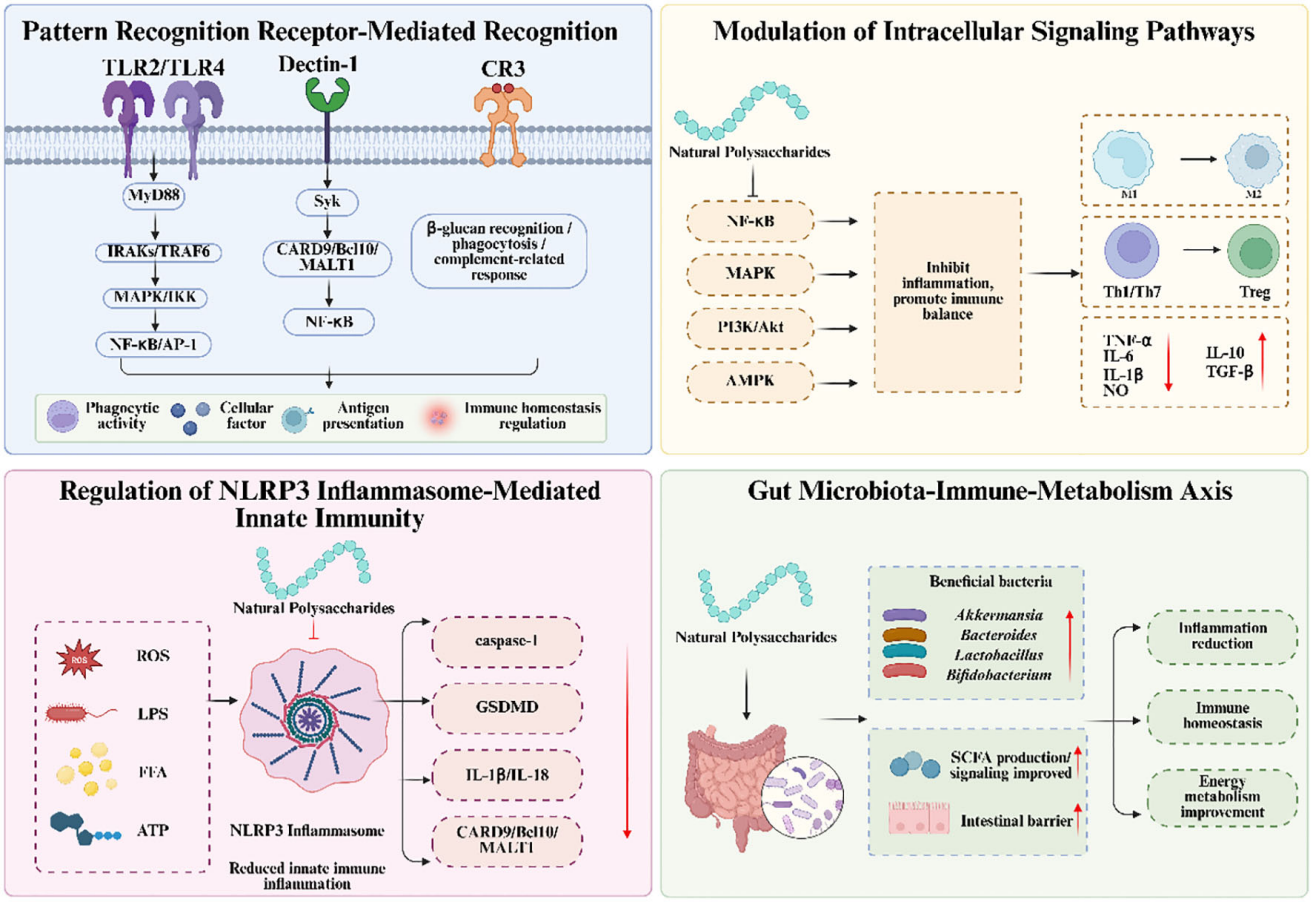
Core mechanism diagram of immune regulation.

Natural polysaccharides regulate metabolic inflammation through multiple interconnected immunological mechanisms. The major mechanisms involve immune recognition through pattern-recognition receptors, regulation of intracellular inflammatory and metabolic signaling networks, suppression of NLRP3 inflammasome activity, and modulation of the gut microbiota-immune-metabolic axis. Through these processes, natural polysaccharides can suppress excessive inflammation, promote immune balance, improve intestinal barrier function, and restore metabolic homeostasis in metabolic diseases (**Figure 3**).

### 4.1 Pattern recognition receptor-mediated recognition

Natural polysaccharides have complex glycan chains and diverse spatial conformations, which allow them to interact with the innate immune system as biological response modifiers (115). However, the principles and underlying mechanisms governing the interactions between polysaccharides and pattern-recognition receptors (PRRs) remain incompletely understood. Their immunomodulatory effects are not simply based on general immune stimulation (116). Instead, these activities are largely mediated by immune-cell receptors that recognize defined polysaccharide motifs and subsequently trigger downstream signaling cascades. PRRs provide a critical molecular basis for polysaccharide-mediated immune regulation. The main PRRs involved include TLRs, complement receptors (CRs), mannose receptor (MR), and C-type lectin receptors (CLRs) (117). These receptors are found in many immune cells, such as macrophages, dendritic cells, intestinal immune cells, and other types of white blood cells. They can recognize different structural types of polysaccharides and activate downstream pathways such as MAPK, NF-κB, and Syk/CARD9 signaling. By activating these signaling routes, polysaccharides regulate cytokine secretion, phagocytic activity, antigen presentation, and immune homeostasis (118, 119). It should be emphasized that the involvement of PRRs in polysaccharide-mediated immunomodulation does not necessarily indicate direct ligand-receptor binding between polysaccharides and these receptors. In many studies, receptor involvement is mainly inferred from changes in receptor expression, activation of downstream signaling pathways, or results obtained using pharmacological inhibitors, whereas direct biophysical evidence demonstrating polysaccharide–receptor interactions remains limited. The evidence supporting polysaccharide recognition by specific receptors is heterogeneous. For some well-characterized polysaccharides, receptor involvement has been examined using binding assays, receptor-blocking strategies, competitive inhibition, or genetic loss-of-function models. However, many studies infer receptor participation mainly from increased receptor expression, downstream phosphorylation events, or changes in inflammatory mediator production. Such results indicate activation of receptor-associated signaling pathways, but they should not be interpreted as definitive evidence of direct polysaccharide–receptor binding. To clarify these distinctions, representative studies are summarized in **Table 2**, with emphasis on the polysaccharide structure, proposed receptor, evidence type, and major limitations.

**Table 2:** Evidence types and limitations supporting proposed receptor involvement in polysaccharide recognition.

Polysaccharide/preparation	Structural characterization	Candidate receptor or receptor-associated pathway	Main experimental evidence	Evidence category	Principal limitation	References
Astragalus polysaccharides or partially purified Astragalus polysaccharide fractions	Heterogeneous plant-derived polysaccharides; molecular weight, monosaccharide composition, glycosidic linkages, and purity vary among studies	TLR4/MyD88/NF-κB or TLR2/4-associated pathways	Changes in the expression or phosphorylation of TLR4, MyD88, NF-κB, MAPKs, and related signaling molecules; altered production of NO, TNF-α, IL-6, and other inflammatory mediators; some studies used pathway inhibitors or receptor-blocking strategies	Mainly downstream signaling evidence; some functional inhibition evidence	Direct binding evidence is lacking in most studies; endotoxin contamination, preparation heterogeneity, and differences in purity may confound TLR-related interpretations	(120–122)
Yeast- or fungal-derived β-glucan preparations	Usually contain a β-(1→3)-glucan backbone with β-(1→6) branches; molecular weight, solubility, particulate state, and higher-order conformation differ among preparations	Dectin-1; CR3 or TLR-related cooperative signaling may be involved in some contexts	Dectin-1 blockade, activation of Syk/CARD9/NF-κB signaling, enhanced phagocytosis, ROS production, and cytokine release; some preparations have binding-related or functional validation evidence	Functional receptor validation and downstream signaling evidence; some preparations have stronger binding-related evidence	Evidence strength depends strongly on β-glucan source, purity, molecular weight, branching pattern, solubility, and particulate state; findings from a specific β-glucan preparation should not be generalized to all natural polysaccharides	(123–125)
Ganoderma lucidum, Lentinus edodes, and other fungal β-glucan-like polysaccharides	Fungal polysaccharides or polysaccharide fractions; β-glycosidic linkages, branching patterns, or triple-helix conformations are often reported, but purity and structural definition vary	Dectin-1, TLR2/4, CR3, and other candidate PRRs	Activation of macrophages or dendritic cells, cytokine release, and changes in Syk, MAPK, and NF-κB signaling; some studies used receptor inhibitors, blocking antibodies, or competitive ligands	Mainly downstream signaling evidence and structure-based inference; some functional validation evidence	Multiple PRRs may be involved simultaneously, and receptor specificity often remains unclear; many studies lack direct binding assays or receptor knockdown/knockout validation	(126, 127)
Mannose-enriched or terminal mannose-containing plant polysaccharides, such as Aloe vera acemannan-related fractions	Contain mannose or acetylated mannose residues; molecular weight, degree of acetylation, and purification level may influence activity	Mannose receptor or other lectin-like receptors	Changes in mannose receptor expression, polysaccharide uptake, colocalization, and activation of macrophages or dendritic cells; some studies used competitive sugars or receptor-blocking strategies	Uptake/colocalization evidence, functional inhibition evidence, or indirect receptor-associated evidence	Mannose-containing structures provide only a plausible structural basis; increased receptor expression or enhanced uptake alone does not prove direct binding	(128)
PGG-glucan or selected soluble β-glucan preparations	Relatively defined or semi-defined β-glucan preparations; structure, solubility, and aggregation state are affected by preparation methods	CR3-associated mechanisms, lactosylceramide-associated receptors, or other β-glucan-binding molecules	Antibody blocking, macrophage activation, concentration-dependent binding, CR3 priming, or iC3b-dependent functional assays	Functional blocking evidence; some studies provide binding-related evidence	Recognition may not be restricted to CR3; receptor identity and functional outcomes may depend on cell type, β-glucan structure, and the experimental system	(129)
Fucoidan and other sulfated marine polysaccharides	Sulfated, negatively charged polysaccharides rich in fucose; molecular weight, degree of sulfation, sugar composition, and purity vary markedly among preparations	Scavenger receptors, TLRs, C-type lectin receptors such as CLEC-2, and selectin-related interactions	Cellular uptake, altered inflammatory signaling, reduced cytokine production, and platelet or immune-cell activation assays; some studies used receptor-deficient or receptor-blocking models	Mainly pathway modulation, uptake evidence, and functional validation evidence; stronger functional evidence exists for selected targets	Degree of sulfation, charge density, molecular weight, and impurities may influence biological effects; different fucoidan preparations should not be considered equivalent	(130, 131)
Alginate, alginate oligosaccharides, and modified alginate derivatives	Marine uronic acid-containing polysaccharides; degradation, oligomerization, or chemical modification can alter solubility, molecular weight, and charge properties	TLR2/4 or other membrane-associated receptor pathways	Changes in inflammatory mediators, oxidative stress markers, NF-κB/MAPK signaling, or gut immune-related responses	Mainly downstream signaling evidence and structure-based inference	Direct receptor-recognition evidence is limited; some effects may be mediated indirectly through gut microbiota, viscosity, bile acid metabolism, or intestinal barrier function rather than direct receptor binding	(132)

Among PRRs, TLR2 and TLR4 have been implicated in polysaccharide-associated immune signaling. Polysaccharides derived from plants, fungi, and animals can regulate the functions of macrophages and dendritic cells through TLR2- or TLR4-mediated pathways. A reported Astragalus polysaccharide preparation has been associated with regulation of the TLR4/MyD88-related pathway in immune-cell models. However, in many studies, this conclusion is mainly based on changes in receptor expression or downstream signaling molecules, rather than direct ligand–receptor binding evidence (133). This uncertainty is particularly important for complex polysaccharide preparations, because residual endotoxin contamination, differences in purity, and structural heterogeneity may also activate TLR-related signaling and complicate the interpretation of receptor specificity. Following APS-induced stimulation of TLR4, downstream adaptor proteins, including MyD88, IRAKs, and TRAF6, are recruited to initiate intracellular signal transduction. This process activates MAPK cascades, the IKK complex, AP-1, and NF-κB, thereby promoting the expression of immune and inflammatory mediators such as TNF-α, IL-1β, IL-6, and nitric oxide (NO). However, activation of TLR signaling does not necessarily indicate a purely pro-inflammatory effect. The final biological outcome depends on the source, monosaccharide composition, molecular weight, glycosidic linkages, purity, dose, and disease microenvironment of the polysaccharide. In metabolic diseases, polysaccharides may cause a mild activation of immune cells, which helps improve host defense and the removal of harmful substances. At the same time, polysaccharides may improve the intestinal barrier and reduce the amount of LPS entering the blood; in this way, excessive activation of TLR4 can be weakened, and chronic low-grade inflammation may be relieved. Therefore, the effect of polysaccharides on TLR signaling is not fixed, but changes with the disease condition and the immune environment.

Besides TLRs, CLRs are also involved in the recognition of polysaccharides. Among these receptors, Dectin-1 has been studied more often, especially in the recognition of β-glucans (134). Dectin-1 mainly recognizes *β*-glucans that contain a *β*-(1→3)-linked main chain and *β*-(1→6)-linked side branches. After Dectin-1 binds to these β-glucans, the activation region inside the cell can recruit spleen tyrosine kinase (Syk). Then, Syk further activates the CARD9-Bcl10-MALT1 signaling complex; this process can lead to NF-κB activation, ROS production, stronger phagocytosis, and increased cytokine release. In some cases, Dectin-1 signaling may also work together with TLR2 or TLR4 signaling, so the immune response may be strengthened or changed. For natural polysaccharides containing glucan-like structures, such as some fungal polysaccharides and certain APS fractions, Dectin-1 may participate in immune recognition. However, current findings are largely derived from structural predictions and alterations in downstream signaling events, while direct evidence confirming receptor binding remains insufficient.

The Mannose receptor (MR) is also important in polysaccharide-mediated immune recognition (116). This receptor can recognize polysaccharide structures containing terminal mannose, fucose, or N-acetylglucosamine residues. It is involved in endocytosis, antigen uptake, and antigen presentation. Because some natural polysaccharides contain relatively high levels of mannose residues, they may affect macrophage and dendritic cell function through MR-mediated recognition. Taking APS as an example, the presence of mannose in its monosaccharide composition provides a possible structural basis for interaction with MR. However, direct evidence for APS-MR binding is still limited. In some studies, the observed upregulation of MR expression may also be an indirect result of cell activation. Therefore, the role of MR in polysaccharide immunomodulation needs further confirmation through methods such as surface plasmon resonance, receptor blocking, and gene knockout models. A combination of complementary approaches is therefore required to distinguish direct receptor binding from indirect receptor-associated responses.

Complement receptors, especially complement receptor 3 (CR3/CD11b/CD18), also contribute to the recognition and immunomodulatory effects of some polysaccharides (135). CR3 has also been reported to recognize specific glucan structures (136). CR3-mediated phagocytosis and degranulation appear to depend on dual ligation within the CR3 domain, involving simultaneous binding to iC3b and glucan-associated recognition sites (137). However, PGG-glucan may be recognized by a receptor that differs from CR3, suggesting that glucan sensing may involve additional receptor-dependent mechanisms (138). PGG-glucan has been reported to activate rat macrophages through concentration-dependent, high-affinity binding. Evidence from antibody-blocking experiments further suggests that lactosylceramide glycosphingolipids may function as potential receptors for PGG-glucan. However, this recognition does not appear to be restricted to a single lactosylceramide species, because variants with different fatty acid chain lengths and degrees of saturation may also mediate PGG-glucan binding.

Future studies should use structurally well-defined and highly purified polysaccharide fractions to clarify receptor specificity. Direct binding assays, such as surface plasmon resonance, isothermal titration calorimetry, microscale thermophoresis, affinity chromatography, and affinity-based pull-down assays, should be combined with functional validation strategies, including receptor-blocking antibodies, competitive ligand assays, receptor knockdown or knockout models, and rescue experiments. In addition, endotoxin removal and polymyxin B controls should be included whenever applicable when evaluating TLR-related responses, because endotoxin contamination may confound the interpretation of polysaccharide-induced TLR activation. Combining these approaches with transcriptomics, proteomics, metabolomics, and receptor-dependent signaling analysis would help distinguish direct polysaccharide-receptor interactions from secondary immune responses and provide a more rigorous basis for mechanistic interpretation.

### 4.2 Modulation of intracellular signaling pathways

After receptor-associated recognition or interaction with immune and epithelial cells, natural polysaccharides may affect intracellular signaling pathways related to inflammation and metabolism. Among them, PI3K/Akt, MAPK, and NF-κB are relatively important pathways; they can connect external stimulation with immune-cell activation, and they also take part in the production of inflammatory mediators. Although these pathways are often discussed separately, they do not function as isolated signaling routes. Instead, they form an interconnected immunometabolic network in which inflammatory signaling, oxidative stress, insulin sensitivity, mitochondrial function, macrophage polarization, and intestinal barrier regulation influence each other. As summarized in **Figure 3**, natural polysaccharides may regulate immune-metabolic homeostasis through PRR-associated recognition, intracellular signaling modulation, NLRP3 inflammasome regulation, and the gut microbiota–immune–metabolism axis.

NF-κB is an important factor that regulates inflammatory responses, and it is closely related to the occurrence and persistence of metabolic inflammation (139). Under metabolic stress, factors such as free fatty acids, endotoxin exposure, and oxidative stress can activate the classical NF-κB pathway. This process involves IκBα phosphorylation and degradation, followed by nuclear translocation of NF-κB p65 and increased transcription of pro-inflammatory cytokines and other inflammatory mediators. In many experimental models, natural polysaccharide treatment has been associated with reduced IκBα phosphorylation, decreased NF-κB p65 nuclear translocation, and lower expression of inflammatory mediators. Nevertheless, NF-κB regulation by polysaccharides should not be interpreted as a uniformly inhibitory process. Depending on receptor engagement, structural motifs, and immune context, some polysaccharides may induce mild immune activation, whereas others mainly suppress pathological inflammatory signaling.

MAPK signaling mainly contains three parts, including ERK, JNK, and p38 pathways; these pathways are related to the expression of inflammatory mediators, cellular responses under stress, oxidative injury, and the development of insulin resistance (140). Among these pathways, JNK signaling is especially related to insulin resistance; it can increase the serine phosphorylation of IRS-1, disturb the downstream signaling of the insulin receptor, and then make insulin resistance more serious (141). Natural polysaccharides may reduce the release of pro-inflammatory factors and improve inflammation in metabolic tissues by inhibiting the overactivation of JNK and p38 MAPK. At the same time, some polysaccharides may also regulate ERK signaling; through this pathway, they can affect macrophage activation, changes in macrophage phenotype, and cytokine secretion (142). Because ERK, JNK, and p38 may have different effects in different cell types and at different disease stages, the effect of polysaccharides on MAPK signaling should not be explained in only one way; it needs to be considered together with the specific cell type, disease model, and experimental condition.

The PI3K/Akt pathway functions as an important regulatory axis connecting immune-cell survival, insulin signal transduction, glucose and lipid metabolism, and inflammatory modulation (143). Natural polysaccharides may regulate insulin sensitivity, immune-cell activation, and macrophage polarization through the PI3K/Akt pathway. However, their effects are influenced by multiple factors, including polysaccharide origin, structural properties, dosage, and the pathological microenvironment. The hypoglycemic actions of dietary plant polysaccharides are mainly mediated through several mechanisms (144). First, they may protect pancreatic β-cell structure and function by reducing oxidative stress, inflammation, lipid accumulation, and glucotoxicity, thereby supporting insulin secretion. Second, they can enhance insulin sensitivity and alleviate insulin resistance. Third, they may regulate key enzymes involved in carbohydrate digestion, glucose absorption, utilization, and metabolism. In addition, dietary plant polysaccharides can regulate signaling pathways, including PI3K/Akt and ERK/JNK/MAPK, and may indirectly improve glucose homeostasis by reshaping the gut microbiota. In immune cells, these signaling changes may alter cytokine production and inflammatory resolution. These changes indicate that polysaccharides may shift the inflammatory microenvironment from a pro-inflammatory state toward a reparative and resolution-oriented phenotype (145).

### 4.3 Inhibition of NLRP3 inflammasome-mediated innate immune inflammation

Inflammasome activation is an important mechanism that sustains chronic low-grade inflammation in metabolic diseases, among which the NLRP3 inflammasome has received the most attention (146). It responds not only to microbial danger signals but also to endogenous stress-related stimuli, including extracellular ATP, crystalline substances, saturated fatty acids, mitochondrial dysfunction, and other metabolic danger signals (147). Once activated, NLRP3 promotes inflammasome complex assembly and caspase-1 activation. This process leads to the maturation and extracellular release of IL-1β and IL-18. In parallel, cleavage of gasdermin D (GSDMD) induces membrane pore formation and pyroptotic cell death. Through these processes, NLRP3 activation can amplify inflammatory responses and contribute to tissue injury under metabolic stress conditions. Thus, excessive NLRP3 inflammasome activation represents a shared innate immune mechanism involved in metabolic inflammation (148, 149).

Natural polysaccharides may regulate NLRP3 inflammasome activation at multiple levels (150). First, they may reduce upstream metabolic and oxidative stress signals that trigger inflammasome activation. For example, polysaccharides with antioxidants or mitochondrial-protective activities may reduce ROS accumulation, preserve mitochondrial function, and limit the release of mitochondrial damage-associated signals, thereby suppressing NLRP3 inflammasome priming and activation. Second, polysaccharides may attenuate the priming stage of inflammasome activation by reducing the expression of NLRP3, pro-IL-1β, and related inflammatory mediators. Through these mechanisms, polysaccharides may reduce inflammasome-related effector responses, including caspase-1 activation and IL-1β/IL-18 maturation. These findings indicate that polysaccharide-mediated regulation of inflammasomes is not limited to a single target but may involve several stages, including upstream danger-signal reduction, transcriptional priming, inflammasome complex assembly, and downstream effector release.

Natural polysaccharides may attenuate activation of the NLRP3/caspase-1/GSDMD signaling axis, thereby limiting the maturation and secretion of IL-18 and IL-1β. This effect can further suppress pyroptotic cell death and reduce inflammation-related injury in metabolically stressed tissues. For example, blocking NLRP3 inflammasome activation has been shown to protect against HFD-induced non-alcoholic fatty liver disease. This protective effect is associated with improved insulin sensitivity, reduced hepatic injury, lower caspase-1 activity, and decreased IL-1β expression compared with wild-type mice (151). Therefore, inhibition of excessive NLRP3/caspase-1/GSDMD signaling represents an important mechanism by which natural polysaccharides may limit innate immune inflammation in metabolic disorders.

### 4.4 Gut microbiota-metabolism axis

The gut microbiota-metabolism axis is an important route through which natural polysaccharides regulate immune and metabolic homeostasis (152). Many natural polysaccharides escape digestion in the upper gastrointestinal tract and subsequently enter the colon, where they can be fermented by resident gut microbes. In this process, polysaccharides can serve as microbial carbon sources and are often associated with changes in microbial composition, microbial metabolism, and host immune responses. Current preclinical evidence suggests that natural polysaccharides may alleviate metabolic inflammation by regulating macrophage polarization, suppressing pro-inflammatory cytokine production, modulating MAPK, NF-κB, AMPK, and related signaling pathways, and being associated with changes in the gut microbiota-immune axis (153). The metabolic and immunological effects of these taxa may differ markedly at the species or strain level and may depend on diet, host background, disease stage, baseline microbiota composition, and the physicochemical structure of the polysaccharide substrate. Therefore, polysaccharide-induced microbiota remodeling should be evaluated not only by changes in taxonomic abundance, but also by functional readouts, including SCFA production, bile acid transformation, mucin metabolism, intestinal barrier integrity, inflammatory markers, and host metabolic phenotypes.

When supported by functional evidence, SCFAs represent important microbial metabolites that link polysaccharide fermentation with host metabolic regulation. They help preserve intestinal homeostasis by strengthening epithelial barrier function, modulating gut hormone release, and maintaining a stable intestinal microenvironment. Mechanistically, SCFAs mainly exert their biological effects by activating G protein-coupled receptors, including GPR41 and GPR43, and by modulating epigenetic processes, particularly through the inhibition of histone deacetylases (HDACs) (154). Through these mechanisms, SCFAs may link polysaccharide fermentation to improved intestinal barrier integrity, immune tolerance, and systemic metabolic homeostasis (155, 156).

Bile acid metabolism is also an important part of this axis. Bile acids can act on receptors such as TGR5 and FXR; through these receptors, they may regulate glucose metabolism, lipid and cholesterol metabolism, insulin sensitivity, fatty acid oxidation, and inflammatory responses (157). Abnormal BA metabolism is closely related to obesity, T2DM, MASLD, and CVD. In addition, gut microbiota can change the composition of BAs and produce secondary BAs; these changes may further affect intestinal permeability, inflammatory responses, cholesterol metabolism, and overall metabolic balance. Previous studies have shown that some hyocholic acid species may improve glucose homeostasis through a specific signaling process related to TGR5 and FXR (158). BAs also help regulate liver metabolism by reducing intrahepatic cholesterol synthesis and hepatic fat accumulation. Disturbances in BA synthesis and secretion may contribute to MASLD progression by affecting lipid transport and inflammatory responses in the liver. Hyodeoxycholic acid has been reported to improve MASLD partly by regulating the gut-liver axis (159). A study found that BAs could mediate intracellular cholesterol transport and promote intestinal cholesterol absorption and Niemann-Pick C1-like 1 (NPC1L1) recycling (160). Activation of BA receptors may reduce cholesterol and triglyceride accumulation, thereby contributing to the prevention of atherosclerosis and coronary heart disease (161). Recent human and translational studies provide clinically relevant evidence linking polysaccharide or fiber intervention, gut microbiota changes, and metabolic outcomes (162). In a 4-month randomized placebo-controlled clinical trial in patients with NAFLD, resistant starch intervention (n = 99) resulted in a 9.08% absolute reduction in intrahepatic triglyceride content (IHTC) compared with the control group (n = 97), and this reduction remained 5.89% after adjustment for weight loss. Mechanistically, serum branched-chain amino acids and the gut microbial species *Bacteroides stercoris* were significantly correlated with IHTC and liver enzymes, and both were reduced by resistant starch supplementation. These findings were further supported by fecal microbiota transplantation and monocolonization experiments in mice, indicating that resistant starch may improve hepatic steatosis through gut microbiome-mediated mechanisms.

It should be noted that current studies on the role of natural polysaccharides in modulating the gut microbiota and improving metabolic diseases are still largely based on correlative evidence. Many studies have reported that polysaccharide intervention is accompanied by concomitant changes in gut microbiota composition, short-chain fatty acid levels, bile acid metabolism, and host metabolic phenotypes. However, these findings do not directly demonstrate that alterations in the gut microbiota are responsible for metabolic improvement. Certain microbial changes may contribute to the beneficial effects of polysaccharides, but they may also arise as secondary consequences of improved host metabolic status or simply represent accompanying changes during disease alleviation. Therefore, when interpreting the immunometabolic regulatory effects of polysaccharides through the gut microbiota, changes in microbial composition should not be directly equated with causal mechanisms without further experimental validation.

## 5 Therapeutic advances in metabolic diseases

**Figure 4:**
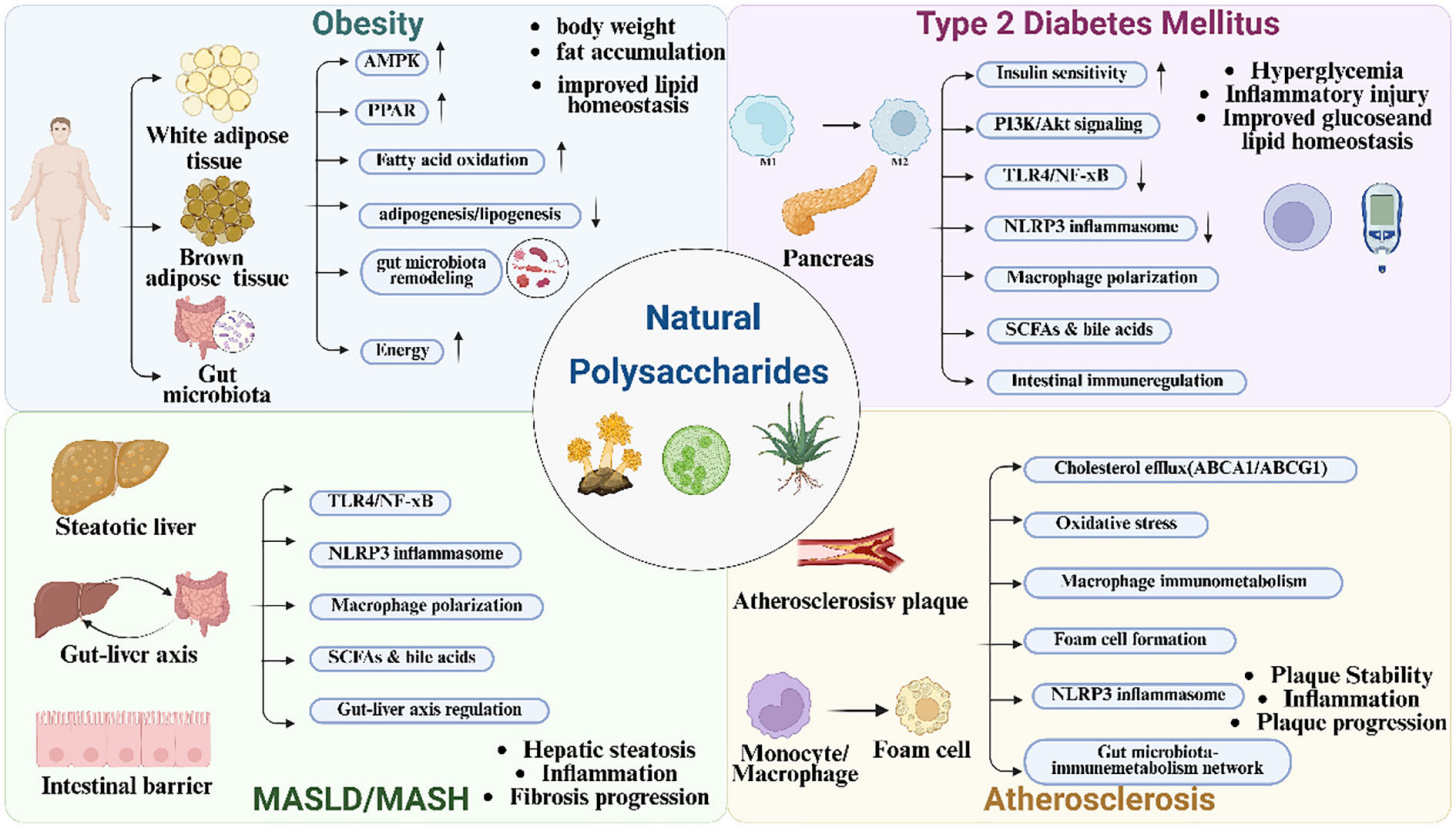
Therapeutic application of natural polysaccharides in metabolic diseases.

Natural polysaccharides may provide therapeutic benefits in obesity, type 2 diabetes mellitus, MASLD/MASH, and atherosclerosis by targeting multiple immunometabolic abnormalities. These effects involve improved lipid handling and insulin sensitivity, rebalanced macrophage polarization, reduced inflammatory pathway activation, inhibited NLRP3 inflammasome signaling, enhanced gut barrier function, and, in some studies, microbiota-associated changes in the gut microbiota-immune-metabolism network (**Figure 4**).

### 5.1 Obesity

The application of natural polysaccharides in obesity management has attracted growing research interest because some preparations have been associated with changes in energy metabolism, lipid homeostasis, and gut microbiota composition, mainly in preclinical obesity models (69). Obesity is now a common health problem in many countries, and it is closely related to several chronic diseases, especially type 2 diabetes and cardiovascular diseases (163). Compared with conventional pharmacological approaches, natural polysaccharides have been investigated as potential non-drug interventions because some preparations show diverse biological activities; however, their safety and efficacy should be evaluated in a preparation-, dose-, and route-specific manner (164). Emerging evidence suggests that specific polysaccharides may help alleviate obesity by influencing key metabolic pathways. However, current research remains at an early stage, and differences in polysaccharide sources, study designs, and experimental models limit direct comparison across studies and make underlying mechanisms difficult to define conclusively.

In animal models, some natural polysaccharide preparations have been reported to reduce fat accumulation in association with altered expression of genes related to lipolysis, fatty acid oxidation, and adipogenesis. For example, *flaxseed* polysaccharide (FP) has been tested mainly in obese animal models and was reported to modulate obesity-related metabolic gene expression, particularly genes associated with fatty acid metabolism, lipolysis, and energy metabolism, including Fabp1-5, Lpl, and Pparg in the PPAR signaling pathway (165, 166). These changes may promote fatty acid oxidation and reduce lipid synthesis. In addition, *Enteromorpha prolifera* polysaccharide (EP) has been tested in high-fat diet-induced obese mice and was reported to activate AMPK-related signaling, improve lipid metabolic disorders, and reduce fat accumulation (167). Its effects may be associated with enhanced lipolysis and suppressed adipogenesis. *Bangia fusco-purpurea* polysaccharide (BFP) has also been reported to alleviate high-fat diet-induced obesity by increasing energy expenditure, promoting fatty acid oxidation, and inhibiting adipogenesis (167).

The gut microbiota may represent an important microbiota-associated component of the anti-obesity effects reported for some natural polysaccharides (168). Because many polysaccharides escape degradation by host digestive enzymes, they can pass into the colon and serve as fermentable substrates for intestinal microbes. This process may reshape gut microbial communities and alter the production of microbial metabolites. Previous studies suggest that *Chlorella pyrenoidosa* polysaccharide (CPS) and *Spirulina platensis* polysaccharide (SPS) can improve high-fat diet-induced gut dysbiosis, enhance hepatic lipolysis, suppress lipogenesis, and reduce fat accumulation (169, 170). FP-derived metabolites may also downregulate the expression of intestinal glucose transporters, such as SGLT1 and GLUT2, thereby reducing glucose absorption. They may further inhibit the expression of adipogenesis-related genes, including *C/EBPβ*, *C/EBPα*, and *PPARγ*, leading to reduced fat formation. *Lyophyllum decastes* polysaccharide has been shown to promote the enrichment of bacteria, including *Bacteroides* and *Lactobacillus johnsonii*, and enhancing brown adipose tissue through secondary bile acids and the TGR5 signaling pathway, showing potential anti-obesity effects (171).

Natural polysaccharides from other sources also show protective effects against obesity. For example, *Lentinus edodes* polysaccharide and *Flos Lonicera* polysaccharides (LF) may limit weight gain and reduce adipose accumulation in animal models of high-fat diet-induced obesity (172). Metabolomic evidence indicates that LF may alleviate obesity-associated metabolic abnormalities by modulating pathways involved in energy homeostasis, choline metabolism, and gut microbiota-derived metabolic processes. In recent years, observational studies have indicated that polysaccharides derived from edible mushrooms possess diverse biological activities, including immunomodulatory effects, anti-obesity potential, and possible anti-aging properties (75). *Ganoderma lucidum* polysaccharides (GLPs) are mainly composed of α- and β-glucans with different glycosidic linkages, such as (1→3) and (1→6) bonds, together with other carbohydrate components that contribute to their biological activities. Current evidence suggests that GLPs may alleviate obesity, modulate immune responses, and exert protective effects against colorectal cancer partly by reshaping gut microbial communities, including *Oscillospira*, *Clostridium* cluster IV, and *Eubacterium* spp (29) (173). These findings indicate that polysaccharides from different sources may regulate obesity through multiple layers, including lipid metabolism, energy expenditure, gut microbiota, and microbial metabolites.

Although most anti-obesity evidence for natural polysaccharides is still derived from animal studies, limited clinical findings have begun to support their translational relevance. A clinical trial was implemented to explore the effects of *Lycium barbarum* polysaccharide-standardized fruit juice on overweight adults (174). The results showed that this fruit juice increased the metabolic rate in a dose-dependent manner. Moreover, the waist circumference of overweight adults was reduced by 5.5 ± 0.8 cm after the 14-day intervention. As noted above, the current clinical trials have shown the beneficial trend of herb-derived polysaccharides in the treatment of obesity. However, no definitive conclusions can be drawn due to the small number of clinical trials and limited sample size.

Overall, natural polysaccharides may have useful potential in the prevention and management of obesity. Their effects are not caused by a single mechanism, but may be related to the regulation of lipid metabolism, the improvement of energy use, the reshaping of gut microbiota, and the increase of microbial metabolites, such as short-chain fatty acids and bile acids. Although a limited clinical study using *Lycium barbarum* polysaccharide-standardized fruit juice reported increased metabolic rate and reduced waist circumference in overweight adults, most current evidence still comes from animal experiments and *in vitro* studies. At the same time, the sources, structures, doses, intervention duration, and experimental models of polysaccharides are different among studies, so the results are not always easy to compare. In future work, the structure-activity relationships of these polysaccharides should be further clarified. The causal roles of gut microbiota and key signaling pathways also need to be verified by methods such as fecal microbiota transplantation, germ-free animal models, receptor blockade, and gene knockout experiments. Before these polysaccharides can be widely used for obesity intervention, their bioavailability, effective dose, long-term safety, and clinical value still need more evaluation in well-designed human studies.

### 5.2 Type 2 diabetes mellitus

Type 2 diabetes mellitus (T2DM) is a common metabolic disease. It is mainly related to impaired insulin secretion and reduced insulin sensitivity in target tissues; because of these changes, the regulation of glucose and lipid metabolism is disturbed, and protein, water, and electrolyte metabolism may also be affected (175). The occurrence and development of T2DM are affected by several factors, such as obesity, genetic susceptibility, impaired islet function, and changes in gut microbiota. These factors are all related to the maintenance of energy metabolism. Among them, insulin resistance is considered an important metabolic risk factor. In the early stage of insulin resistance, pancreatic β cells usually increase insulin secretion to help maintain blood glucose balance. As a result, hyperinsulinemia may gradually appear. However, when β cells can no longer keep this compensatory effect, glucose regulation becomes impaired, and T2DM may finally occur (176). Abdominal obesity is also an important factor related to T2DM (177). Besides this, patients with T2DM usually have different intestinal microbial communities when compared with healthy people, which indicates that gut microbiota disorder may be involved in the progression of this disease. Some animal studies also found abnormal fat accumulation in the liver of T2DM models (178). Suzuki et al. proposed a possible process of disease development, which may begin with weight gain, then increased liver enzymes, and later lead to hypertriglyceridemia (HTG) and impaired glucose tolerance (179).

In recent years, natural polysaccharides have received attention as possible agents for T2DM intervention because some preparations can affect multiple biological targets. However, their clinical use requires careful safety assessment, as tolerability and toxicity may vary according to preparation quality, route of administration, dose, intervention duration, patient background, and concomitant medications. This is mainly because they have good biocompatibility, relatively low toxicity, and can act on several biological targets at the same time (79). Accumulating studies have demonstrated that polysaccharides can alleviate metabolic inflammation through modulation of immune cell function (180). For example, polysaccharides derived from *Grifola frondosa* were shown to reduce hepatic inflammatory responses and improve insulin resistance by suppressing macrophage infiltration and promoting anti-inflammatory macrophage polarization in diabetic models (181). In addition, polysaccharides can regulate T cell-mediated immune responses. *Lycium barbarum* polysaccharides were reported to modulate bile acid metabolism and activate the FXR-FGF15-FGFR4 signaling axis, thereby promoting Treg expansion and suppressing inflammatory responses in diabetic rats (182).

At the molecular level, suppression of inflammatory signaling pathways and inflammasome activation represents another major mechanism underlying the anti-diabetic effects of natural polysaccharides (183). *Astragalus polysaccharides* may alleviate diabetes-related complications by blocking TLR4/NF-κB pathway activation and weakening NLRP3 inflammasome-mediated inflammatory responses, thereby reducing tissue injury (133). Moreover, polysaccharides can improve insulin sensitivity through modulation of key metabolic pathways, including IRS/PI3K/Akt and AGE-RAGE signaling pathways, ultimately contributing to the restoration of glucose and lipid metabolic homeostasis. Together, these results indicate that polysaccharides may produce anti-diabetic effects by jointly modulating inflammatory responses and metabolism-related signaling pathways.

The gut microbiota-immune network has increasingly been viewed as an important pathway through which polysaccharides confer protective effects against T2DM (184, 185). Polysaccharides can serve as fermentable nutrients for intestinal microbes and are converted into short-chain fatty acids, which modulate host immunity and energy homeostasis largely through G protein-coupled receptors, including GPR41, GPR43, and GPR109A (152, 186). Moreover, polysaccharides may improve insulin sensitivity and suppress systemic low-grade inflammation by modulating gut microbial communities, strengthening the intestinal barrier, and limiting endotoxin translocation. Microbial metabolites, including SCFAs and bile acids, can also participate in this process by regulating immune cell functions and maintaining metabolic balance, reflecting the close crosstalk among polysaccharides, intestinal microbiota, and host immune responses (187).

Although substantial preclinical studies have proved that herb-derived polysaccharides may be a promising agent for the treatment of metabolic diseases, few clinical trials have been conducted to date. In one prospective, randomized, double-blind study, researchers evaluated the glucose-lowering and lipid-modulating effects of LBP in individuals with T2DM (188). The findings of this controlled clinical trial indicated that LBP could serve as a potential adjunctive therapy for T2DM, since three months of supplementation led to notable reductions in serum glucose, along with improvements in the insulinogenic index and high-density lipoprotein cholesterol levels. More recently, a systematic review and meta-analysis further supported the possible regulatory role of LBP in glucose and lipid homeostasis. This analysis reported that daily LBP consumption was associated with significant changes in triglycerides, fasting blood glucose (FBG), low-density lipoprotein cholesterol, and high-density lipoprotein cholesterol (189). In another study, Rashid et al. investigated fenugreek polysaccharide in newly diagnosed T2DM patients using a 12-week randomized, placebo-controlled design (190). Their results showed marked decreases in FBG, hemoglobin A1c (HbA1c), total cholesterol, triglycerides, and LDL levels. Taken together, these clinical observations suggest that herb-derived polysaccharides may contribute to the management of T2DM. Nevertheless, additional well-designed clinical trials with larger sample sizes are still required to verify their therapeutic effectiveness in human populations.

### 5.3 MASLD/MASH

Metabolic dysfunction-associated steatohepatitis (MASH) is a progressive chronic liver disorder characterized by hepatic lipid deposition, inflammatory responses, and hepatocellular injury (191). It is mainly characterized by hepatocyte damage, inflammatory response, and fibrosis (192). The main pathological features of MASH include hepatic lipid deposition, lobular inflammatory infiltration, hepatocellular damage or death, and progressive fibrotic remodeling (193). Accumulating studies indicate that MASH is strongly associated with metabolic disorders, particularly obesity, diabetes, and metabolic syndrome (194). Among these metabolic abnormalities, T2DM is regarded as a major contributor to MASH development and is strongly associated with unfavorable clinical prognosis (195). Epidemiological evidence indicates that metabolic comorbidities are highly prevalent among patients with MASH, with hyperlipidemia, obesity, and T2DM reported in approximately 83%, 82%, and 48% of cases, respectively (196). As the disease continues to develop, MASH may further progress to cirrhosis or end-stage liver disease, and some patients may finally need liver transplantation. MASH is also related to an increased risk of liver-related death and overall death, so early diagnosis and proper intervention are important for improving patient outcomes (197).

Hepatic macrophages are key regulators in MASLD, and changes in their activation state and polarization phenotype can substantially influence disease progression (198). In response to lipotoxic stimuli, oxidative stress, and endotoxin challenge, Kupffer cells together with monocyte-derived macrophages tend to shift toward a pro-inflammatory phenotype, leading to increased production of IL-1β, TNF-α, IL-6, MCP-1, and other inflammatory mediators (66, 199). These cytokines further promote hepatic lipid accumulation, hepatocyte injury, and hepatic stellate cell activation. Continuous activation of the TLR4/NF-κB pathway and NLRP3 inflammasome strengthens hepatic inflammatory signaling and facilitates the progression from lipotoxic injury to inflammation-driven fibrosis (200). Therefore, targeting macrophage polarization and inflammatory signaling pathways has become an important strategy for MASLD/MASH treatment.

Growing evidence suggests that natural polysaccharides may slow the progression of MASLD by regulating immune homeostasis within the liver microenvironment (167). Plant-derived polysaccharides exhibit potent immunomodulatory and immune-regulatory activities by inhibiting NF-κB, MAPK, and NLRP3-associated pathways, which decreases pro-inflammatory cytokine release and facilitates macrophage shift toward an M2-like anti-inflammatory phenotype. For example, GP-derived polysaccharides may alleviate inflammatory responses by modulating the TLR2/NLRP3 signaling axis (201). In a mouse model of MASH, GP administration markedly downregulated TLR-related genes, including TLR1, TLR2, and TLR4, lowered NLRP3 expression, and suppressed the transcription of pro-inflammatory genes. Li et at (202) reported that CP alleviated hepatic inflammation in MASLD rats by suppressing *Irf1* expression, a key regulator involved in hepatitis-related inflammatory responses.

The gut-liver axis refers to the dynamic communication among intestinal microbiota, the gut barrier, and the liver, and maintaining its balance is essential for human health (203). Polysaccharides may improve MASLD by acting on different parts of the gut-liver axis. Studies show that polysaccharides from *Astragalus membranaceus* and *Ophiopogon* polysaccharide (MDG-1) can help restore the balance of gut microbiota and increase beneficial bacteria, which may improve lipid metabolism and reduce fat accumulation in the liver (204, 205). Polysaccharides from *Poria cocos* and *Artemisia sphaerocephala* can strengthen the intestinal barrier, reduce intestinal permeability, and limit the movement of LPS and other endotoxins into the blood, thereby reducing liver inflammation (206, 207). In addition, polysaccharides can be fermented by gut bacteria, and this process produces SCFAs, including acetate, propionate, and butyrate (208). These metabolites can help protect the intestinal barrier, take part in lipid metabolism, and reduce inflammatory responses. Some polysaccharides may also affect bile acid metabolism by regulating pathways related to CYP7A1, CYP8B1, and FXR-SHP/FGF, and this effect may help inhibit fatty acid synthesis and improve fat accumulation in the liver (209). Although most evidence regarding polysaccharide-based interventions in MASLD/MASH is still derived from preclinical models, limited clinical findings have begun to support their translational relevance. Xing et al. conducted a randomized and controlled clinical trial to evaluate the effects of *Lycium barbarum* glycopeptide capsules on liver function and blood lipid levels in patients with NAFLD (210). The results indicated that treatment with *Lycium barbarum* glycopeptide (60 mg/d) for 6 months remarkably reduced the levels of ALT, AST, γ-glutamyl transpeptidase (GGT), TC, TG and LDL-C, and significantly increased HDL-C levels. Additionally, the efficacy of 6-month treatment was better than that of 3-month treatment, indicating that the duration of the intervention is one of the key factors affecting its therapeutic efficacy.

### 5.4 Atherosclerosis

Atherosclerosis is a chronic disease related to both inflammation and metabolic disorder, and it is still an important cause of cardiovascular disease burden around the world (211). Macrophages play an important role in this process (212). When circulating monocytes move into the vascular intima, they can change into macrophages. These macrophages then take up oxidized low-density lipoprotein and slowly become foam cells, which promotes the enlargement of the lipid core and the development of plaques (213). At the same time, macrophages can secrete inflammatory cytokines, including TNF-α, IL-1β, and IL-6, and these factors may further worsen endothelial damage and local inflammation. Therefore, AS is not only considered a disease of lipid deposition, but also an immunometabolic disease related to macrophage dysfunction and long-term inflammation (212).

The metabolic changes of macrophages also have an important effect on the progression of AS (214). Pro-inflammatory M1-like macrophages mainly depend on increased glycolysis, and this process is usually accompanied by more ROS production and inflammatory cytokine release. For example, after oral treatment with Poria cocos polysaccharides (PCPs), the serum levels of TNF-α, IL-6, NO, LDL-C, TG, and TC were reduced in high-fat diet-fed Apoe^−/−^ mice. In the same model, PCPs also showed some antioxidant effects, as they decreased malondialdehyde (MDA) levels and increased superoxide dismutase (SOD) activity. Further mechanistic results showed that PCPs could inhibit the TLR4/NF-κB pathway, which reduced the production of inflammatory mediators and helped improve abnormal lipid metabolism (100). Based on these effects, PCPs may improve metabolic disorders such as MASLD and AS through several related ways, including regulating metabolic pathways, reducing inflammatory responses, and weakening oxidative stress. These changes can promote foam cell formation, oxidative stress, and plaque instability. By contrast, M2-like macrophages use more oxidative phosphorylation and fatty acid oxidation, and they are more related to anti-inflammatory effects, tissue repair, cholesterol efflux, and plaque stabilization (215, 216). Therefore, the balance between M1-like and M2-like macrophages is closely linked to the development and stability of plaques.

Lipid metabolic disorder is also an important event in AS mediated by macrophages (217). In plaques, macrophages can take up more lipids through scavenger receptors, such as CD36 and SR-A1, while cholesterol efflux controlled by ABCA1 and ABCG1 is often weakened. Because of this imbalance, too much cholesterol accumulates inside the cells, and foam cells are gradually formed. In addition, mitochondrial dysfunction and excessive ROS production may activate the NLRP3 inflammasome, then promote the release of IL-1β and IL-18, and induce macrophage pyroptosis or apoptosis. These changes can further enlarge the necrotic core and increase the possibility of plaque rupture (212).

Natural polysaccharides may exert anti-atherosclerotic effects through multiple pathways (218). Their effects are not limited to lipid lowering, but may also involve regulating macrophage polarization, inhibiting inflammatory signaling pathways, reducing oxidative stress, promoting cholesterol efflux, and improving the gut microbiota-immune-metabolism network (219, 220). Some polysaccharides can also act as prebiotic substrates and be fermented by gut microbiota to produce short-chain fatty acids, which further regulate immune and metabolic responses.

Although most evidence supporting the anti-atherosclerotic effects of natural polysaccharides still comes from animal and cellular models, several human studies on polysaccharide-rich or polysaccharide-based interventions provide indirect but clinically relevant evidence by improving atherogenic lipid profiles. For example, a meta-analysis of randomized controlled trials in hypercholesterolemic adults showed that oat β-glucan significantly reduced total cholesterol by 0.24 mmol/L and LDL-C by 0.27 mmol/L, although no significant effects were observed on TG or HDL-C (221). Similarly, konjac glucomannan, a viscous soluble polysaccharide, was reported to reduce LDL-C by 0.35 mmol/L and non-HDL-C by 0.32 mmol/L in randomized controlled trials, corresponding to approximate reductions of 10% and 7%, respectively (222). In addition, recent clinical evidence suggests that sodium alginate may lower serum TC and LDL-C levels in patients with hypercholesterolemia. These findings suggest that certain viscous or marine-derived polysaccharides may contribute to atherosclerosis risk reduction mainly through lipid-lowering effects. However, direct clinical evidence demonstrating plaque regression, reduced carotid intima-media thickness, or fewer cardiovascular events after polysaccharide intervention remains insufficient.

## 6 Challenges and future perspectives

**Figure 5:**
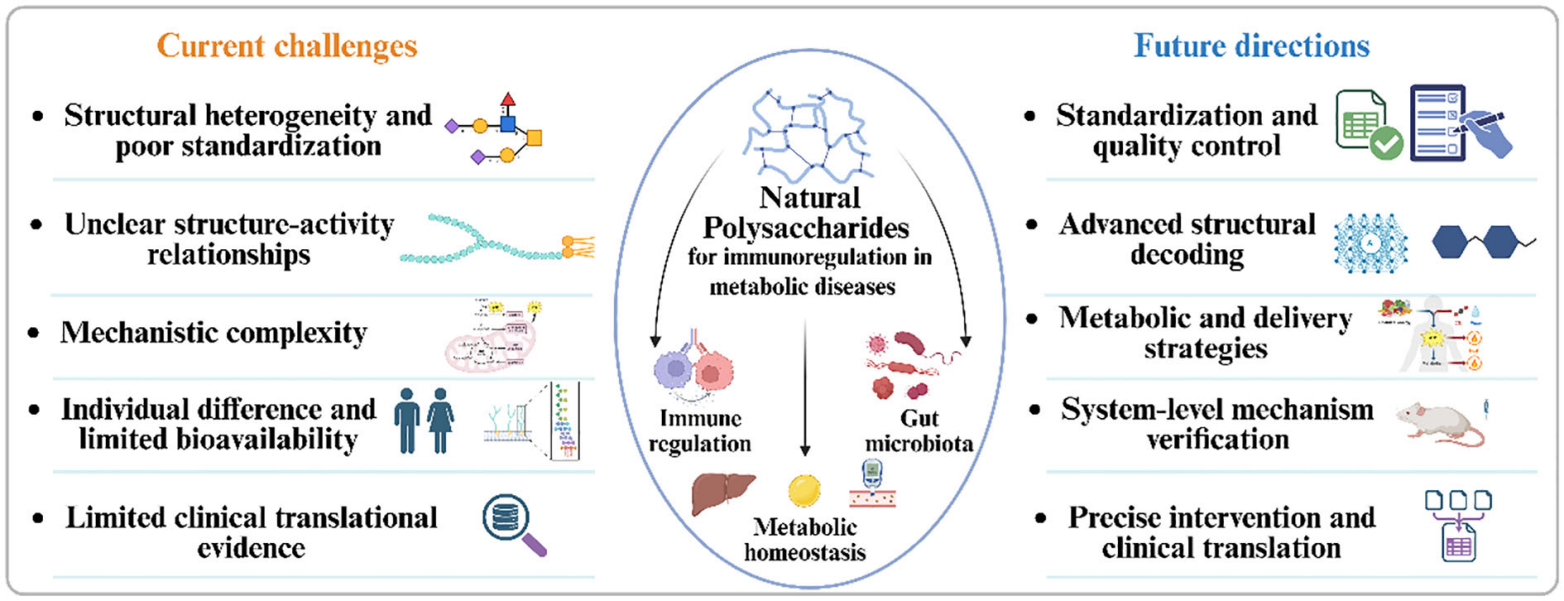
Challenges and future directions diagram.

Although natural polysaccharides show promising potential for immunomodulatory intervention in metabolic diseases, several challenges still limit their clinical application (**Figure 5**). First, natural polysaccharides have complex structures and are easily affected by their biological source, geographic origin, extraction method, purification procedure, and storage conditions. Across different studies, polysaccharides may show marked structural differences, such as variations in molecular weight, sugar composition, linkage types, branching degree, and spatial configuration. These differences may cause the experimental results to be inconsistent. At present, crude extracts or partly purified polysaccharide fractions are still used in many studies, while standard preparation methods and quality control systems are not well established. Because of this, the repeatability of related findings is reduced, and the further development and application of polysaccharides are also limited.

The biological activities of polysaccharides are closely related to their structural features. For example, the triple-helix structure of β-glucans is usually thought to be helpful for their binding with receptors on immune cells. Some chemical modifications, such as carboxymethylation and sulfation, can change the charge distribution, water solubility, and spatial structure of polysaccharides, and these changes may further affect their immunomodulatory effects. Besides this, molecular weight, branching degree, glycosidic linkage type, and functional groups may also influence receptor binding, cell uptake, and related downstream signaling. However, the structure-activity relationship of polysaccharides is still not clear enough. Many studies only report the association between structural characteristics and biological activities, but the specific structural parts that play key roles in immune regulation are still uncertain. This problem is more obvious in complex heteropolysaccharides, so further studies are still needed.

Second, the absorption and metabolic process of natural polysaccharides in the body are still not fully clear. After oral administration, most polysaccharides are not easily absorbed in their complete form, mainly because they usually have a high molecular weight and strong hydrophilicity, which makes it difficult for them to pass through the intestinal epithelium. Because of this, their degradation, fermentation, transformation, and excretion in the gastrointestinal tract become relatively complex. It should also be noted that the effects of polysaccharides may not completely depend on the absorption of intact molecules into the whole body. Many polysaccharides can be fermented by intestinal microorganisms, and this process can produce metabolites, such as short-chain fatty acids and bile acid-related compounds. These metabolites may further regulate host immunity and energy metabolism. However, because the composition of gut microbiota differs among individuals, the responses to polysaccharide intervention may also be different. Therefore, more studies are still needed to clarify the tissue distribution, metabolic fate, long-term safety, and possible interactions between polysaccharides and drugs or other nutrients.

From the mechanistic view, natural polysaccharides usually do not work through only one target, but may produce effects through several pathways. Previous studies have shown that polysaccharides can regulate many immune-related signaling networks, including TLR2/4- and Dectin-1-mediated recognition, NF-κB, MAPK, PI3K/Akt signaling, and NLRP3 inflammasome activation. These pathways are also related to oxidative stress, mitochondrial function, intestinal barrier function, and lipid metabolism. Through these immunometabolic mechanisms, polysaccharides may have beneficial effects on metabolic disorders, such as obesity, insulin resistance, MASLD/MASH, and atherosclerosis. For example, they may regulate macrophage phenotypes, inhibit inflammasome activation, restore the balance of gut microbiota, and reduce long-term low-grade inflammation. However, many current studies still pay more attention to a single signaling pathway, while the wider relationships among immune cells, metabolic tissues, and gut microbiota are not well understood. In addition, many conclusions mainly come from animal experiments or *in vitro* cell models, so whether these findings can be applied to human diseases still needs further confirmation.

The lack of strong clinical evidence is also an important limitation. Although many preclinical studies have shown that natural polysaccharides may improve insulin resistance, reduce inflammation in adipose tissue, regulate blood lipid levels, and decrease lipid accumulation in the liver, the current clinical evidence is still not enough. Most existing clinical studies are limited by small sample sizes, short intervention periods, and differences in polysaccharide sources and doses. To distinguish preclinical findings from clinical evidence, available human studies of polysaccharide-based interventions in metabolic disease-related populations are summarized in **Table 3**. In addition, the long-term safety, effective dose range, possible interactions with drugs, and suitable populations for polysaccharide use are still unclear. Therefore, future studies should establish more standardized quality control and efficacy evaluation systems. Large-scale randomized controlled trials with longer follow-up periods are still required to clarify the clinical relevance of natural polysaccharides for preventing and managing metabolic diseases.

**Table 3:** Typical human studies on the effects of polysaccharide intervention in metabolic diseases.

Preparation	Source	Study design	Population	Sample size	Dose	Duration	Comparator	Endpoints	Adverse events	Major limitations	References
Oat Beta-Glucan	endospermic cell wall of oats	randomized controlled trial	patients aged over 18 years old with hypercholesterolemia	927	1.5 to 6 g/d	3 to 8wk	bread, rice, soup, and a diet without oat beta-glucan or oats	reduce the level of TC and LDL-c	may have led to changes in lipid profiles in hypercholesterolemic patients	need more large-scale, high-quality randomized controlled trials	(221)
Fucoidan	fucoidan extract	Single-site, double-blinded, placebo-controlled, randomized controlled trial	obese 18 and 65 years	72	500 mgtwice daily	90 days	placebo capsules	did not markedly affect other measured parameters of cardiometabolic health in an obese	Not reported	an intrinsic lack of efficacy, lower than measured adherence	(93)
Yeast β-glucan	dietary fiber	a randomized, placebo-controlled, two-arm phase I exploratory intervention	18 and 85 years male and female participants	39	2.5 g/d	8 weeks	maltodextrin as a placebo control	ameliorated hyperinsulinemia and insulin resistance	both yeast β-glucan and the maltodextrin placebo were not identical products and thus this was not a blinded intervention.	The limited sample size used in this current study aimed to determine significance in the primary outcome measure (microbiota composition).	(86)
Lycium barbarum polysaccharide	Lycium barbarum (wolf berry/goji berry)	a randomized, controlled clinical trial	type2 diabetes	67	300mg/day body weight	3 months	placebo capsule	Serum glucose decreased significantly and insulinogenic index increased	LBP efficacy was not obvious efficacy in patients taking hypoglycemic medicines.	The interventional time and dose are small, and this may have led to weaker associations for LBP with some of the variables, such as TG, TC, ApoB and so on.	(188)
konjac glucomannan	soluble fiber	randomized controlled trial	adults and children	370	2.0 to 15.1 g/d	3to 12 wk	cornstarch, wheat bran, maltodextrin	LDL cholesterol (−10%)	gastrointestinal discomfort	optimal dosage remains undetermined	(222)

With the development of glycomics, metabolomics, single-cell sequencing, and AI-assisted structural analysis, research on natural polysaccharides is expected to become more precise. Future studies should strengthen both structural characterization and functional validation, and clarify the relationships among polysaccharide structure, gut microbiota changes, immune cell status, and metabolic improvement. At the same time, nanodelivery systems, chemical modification, and engineered polysaccharides may improve the stability, targeting ability, and application efficiency of polysaccharides. For example, nanodelivery may help polysaccharides reach the gut or specific tissues more effectively, reduce degradation, and enhance their anti-inflammatory and immunomodulatory effects. Considering the large individual differences in gut microbiota and metabolic status, more personalized polysaccharide interventions based on individual characteristics may also become an important direction in the future. In addition to mechanistic and clinical issues, future studies should also consider the sustainability of polysaccharide production. Life-cycle thinking, resource-efficient extraction, waste reduction, and ecosystem-based natural resource management may help improve the environmental and socio-economic value of natural polysaccharide products. Integrating these perspectives will support the responsible development of natural polysaccharides for metabolic disease prevention and sustainable food-health systems.

## 7 Conclusion

Chronic low-grade inflammation, immune dysregulation, and abnormalities in glucose and lipid metabolism are closely associated with the onset and progression of metabolic diseases. Established findings from cell-based and animal studies indicate that natural polysaccharides from plants, fungi, and marine organisms can modulate several immunometabolic processes, including inflammatory cytokine production, macrophage functional states, intracellular inflammatory signaling, inflammasome activation, gut microbiota composition, and microbial metabolite production. These findings suggest that natural polysaccharides may influence the interaction between immune regulation and metabolic homeostasis under experimental conditions. However, several important questions remain unresolved. The current evidence base is still predominantly preclinical, and available clinical studies are limited and heterogeneous. Therefore, the reported biological effects should not be directly interpreted as evidence of clinical efficacy or general safety. In addition, natural polysaccharide preparations differ greatly in source, extraction method, purity, molecular weight, monosaccharide composition, glycosidic linkage pattern, branching structure, dose, and route of administration. These differences make it difficult to compare results across studies and to define reliable structure-activity relationships. The absorption, degradation, fermentation, transformation, and metabolic fate of many polysaccharides *in vivo* also remain incompletely understood. Moreover, it is often unclear whether observed metabolic improvements are caused by direct immune regulation, gut microbiota-mediated effects, or secondary changes following disease alleviation. Future studies should therefore focus on well-characterized polysaccharide preparations and rigorous mechanistic validation. Detailed structural analysis, standardized extraction and purification procedures, quality-control criteria, and batch-to-batch consistency are needed to improve reproducibility. Causal experiments using receptor-blocking strategies, genetic models, microbiota-depletion or transplantation approaches, and multi-omics analyses may help clarify direct and indirect mechanisms of action. In addition, pharmacokinetic behavior, bioavailability, optimal dose, long-term safety, and target populations should be evaluated in well-designed human studies. Overall, current evidence supports further investigation of natural polysaccharides as immunometabolic modulators, but their clinical value remains to be established through standardized preparations and high-quality clinical validation.

## References

[1] Newgard CB . Metabolomics and metabolic diseases: where do we stand? Cell Metab. (2017) 25:43–56. doi: 10.1016/j.cmet.2016.09.018 28094011 PMC5245686

[2] Kojta I Chacińska M Błachnio-Zabielska A . Obesity, bioactive lipids, and adipose tissue inflammation in insulin resistance. Nutrients. (2020) 12:1305. doi: 10.3390/nu12051305 32375231 PMC7284998

[3] Zatterale F Longo M Naderi J Raciti GA Desiderio A Miele C . Chronic adipose tissue inflammation linking obesity to insulin resistance and type 2 diabetes. Front Physiol. (2020) 10:1607. doi: 10.3389/fphys.2019.01607 32063863 PMC7000657

[4] Li H Meng Y He S Tan X Zhang Y Zhang X . Macrophages, chronic inflammation, and insulin resistance. Cells. (2022) 11:3001. doi: 10.3390/cells11193001 36230963 PMC9562180

[5] Hotamisligil GS . Inflammation, metaflammation and immunometabolic disorders. Nature. (2017) 542:177–85. doi: 10.1038/nature21363 28179656

[6] Liu R Nikolajczyk BS . Tissue immune cells fuel obesity-associated inflammation in adipose tissue and beyond. Front Immunol. (2019) 10:1587. doi: 10.3389/fimmu.2019.01587 31379820 PMC6653202

[7] Catrysse L van Loo G . Inflammation and the metabolic syndrome: the tissue-specific functions of NF-κB. Trends Cell Biol. (2017) 27:417–29. doi: 10.1016/j.tcb.2017.01.006 28237661

[8] Zmora N Bashiardes S Levy M Elinav E . The role of the immune system in metabolic health and disease. Cell Metab. (2017) 25:506–21. doi: 10.1016/j.cmet.2017.02.006 28273474

[9] Dias TR Bernardino RL Meneses MJ Sousa M Sá R Alves MG . Emerging potential of natural products as an alternative strategy to pharmacological agents used against metabolic disorders. Curr Drug Metab. (2016) 17:582–97. doi: 10.2174/1389200217666160229113629 26923396

[10] Hotamisligil GS . Inflammation and metabolic disorders. Nature. (2006) 444:860–7. doi: 10.1038/nature05485 17167474

[11] Zhou W Liu P Xu W Ran L Yan Y Lu L . A purified fraction of polysaccharides from the fruits of Lycium barbarum L. improves glucose homeostasis and intestinal barrier function in high-fat diet-fed mice. Food Funct. (2023) 14:5311–25. doi: 10.1039/d3fo00262d 37203380

[12] Zhang Y Lu J Li H Song H . Advances in dietary polysaccharides as hypoglycemic agents: mechanisms, structural characteristics, and innovative applications. Crit Rev Food Sci Nutr. (2025) 65:1383–403. doi: 10.1080/10408398.2023.2293254 38095578

[13] Xie JH Jin ML Morris GA Zha XQ Chen HQ Yi Y . Advances on bioactive polysaccharides from medicinal plants. Crit Rev Food Sci Nutr. (2016) 56:S60–84. doi: 10.1080/10408398.2015.1069255 26463231

[14] Chen R Xu J Wu W Wen Y Lu S El-Seedi HR . Structure–immunomodulatory activity relationships of dietary polysaccharides. Curr Res Food Sci. (2022) 5:1330–41. doi: 10.1016/j.crfs.2022.08.016 36082139 PMC9445227

[15] Nam J Kim A Kim K Moon JH Baig J Phoo M . Engineered polysaccharides for controlling innate and adaptive immune responses. Nat Rev Bioeng. (2024) 2:733–51. doi: 10.1038/s44222-024-00193-2 41059472 PMC12499632

[16] Shen Y Zhao H Wang X Wu S Wang Y Wang C . Unraveling the web of defense: the crucial role of polysaccharides in immunity. Front Immunol. (2024) 15:1406213. doi: 10.3389/fimmu.2024.1406213 39524445 PMC11543477

[17] Ferreira SS Passos CP Madureira P Vilanova M Coimbra MA . Structure–function relationships of immunostimulatory polysaccharides: a review. Carbohydr Polym. (2015) 132:378–96. doi: 10.1016/j.carbpol.2015.05.079 26256362

[18] Duan Y Huang J Sun M Jiang Y Wang S Wang L . Poria cocos polysaccharide improves intestinal barrier function and maintains intestinal homeostasis in mice. Int J Biol Macromol. (2023) 249:125953. doi: 10.1016/j.ijbiomac.2023.125953 37517750

[19] Peng Y Zhao L Hu K Yang Y Ma J Zhai Y . Anti-fatigue effects of Lycium barbarum polysaccharide and effervescent tablets by regulating oxidative stress and energy metabolism in rats. Int J Mol Sci. (2022) 23:10920. doi: 10.3390/ijms231810920 36142831 PMC9504225

[20] López-Otín C Blasco MA Partridge L Serrano M Kroemer G . Hallmarks of aging: an expanding universe. Cell. (2023) 186:243–78. doi: 10.1016/j.cell.2022.11.001 36599349

[21] Zhong RF Liu CJ Hao KX Fan XD Jiang JG . Polysaccharides from Flos Sophorae Immaturus ameliorates insulin resistance in IR-HepG2 cells by co-regulating signaling pathways of AMPK and IRS-1/PI3K/AKT. Int J Biol Macromol. (2024) 280:136088. doi: 10.1016/j.ijbiomac.2024.136088 39366625

[22] Lin F Yu W Li P Tang S Ouyang Y Huang L . Polygonatum sibiricum polysaccharides enhance pancreatic β-cell function in diabetic zebrafish by mitigating mitochondrial oxidative damage via the AMPK-SIRT1 pathway. Front Nutr. (2025) 12:1601490. doi: 10.3389/fnut.2025.1601490 40458823 PMC12128605

[23] Zhang Y Cao Y Wang F Wang L Xiong L Shen X . Polysaccharide from Momordica charantia L. alleviates type 2 diabetes mellitus in mice by activating the IRS1/PI3K/Akt and AMPK signaling pathways and regulating the gut microbiota. J Agric Food Chem. (2025) 73:7298–309. doi: 10.1021/acs.jafc.4c12660 40085053

[24] Jiang Q Chen L Wang R Chen Y Deng S Shen G . Hypoglycemic mechanism of Tegillarca granosa polysaccharides on type 2 diabetic mice by altering gut microbiota and regulating the PI3K-akt signaling pathway. Food Sci Hum Wellness. (2024) 13:842–55. doi: 10.26599/fshw.2022.9250072

[25] Ho Do M Seo YS Park H-Y . Polysaccharides: bowel health and gut microbiota. Crit Rev Food Sci Nutr. (2021) 61:1212–24. doi: 10.1080/10408398.2020.1755949 32319786

[26] Tiwari UP Singh AK Jha R . Fermentation characteristics of resistant starch, arabinoxylan, and β-glucan and their effects on the gut microbial ecology of pigs: a review. Anim Nutr. (2019) 5:217–26. doi: 10.1007/978-981-99-3997-8_6 31528722 PMC6737498

[27] Zhao T Wang C Liu Y Li B Shao M Zhao W . The role of polysaccharides in immune regulation through gut microbiota: mechanisms and implications. Front Immunol. (2025) 16:1555414. doi: 10.3389/fimmu.2025.1555414 40230839 PMC11994737

[28] Zhou YF Nie J Shi C Zheng WW Ning K Kang J . Lysimachia christinae polysaccharide attenuates diet-induced hyperlipidemia via modulating gut microbes-mediated FXR–FGF15 signaling pathway. Int J Biol Macromol. (2023) 248:125725. doi: 10.1016/j.ijbiomac.2023.125725 37419267

[29] Dai H Shan Z Shi L Duan Y An Y He C . Mulberry leaf polysaccharides ameliorate glucose and lipid metabolism disorders via the gut microbiota-bile acids metabolic pathway. Int J Biol Macromol. (2024) 282:136876. doi: 10.1016/j.ijbiomac.2024.136876 39490871

[30] Russo L Lumeng CN . Properties and functions of adipose tissue macrophages in obesity. Immunology. (2018) 155:407–17. doi: 10.1111/imm.13002 30229891 PMC6230999

[31] Chavakis T Alexaki VI Ferrante AW Jr Jr . Macrophage function in adipose tissue homeostasis and metabolic inflammation. Nat Immunol. (2023) 24:757–66. doi: 10.1038/s41590-023-01479-0 37012544

[32] Yan J Horng T . Lipid metabolism in regulation of macrophage functions. Trends Cell Biol. (2020) 30:979–89. doi: 10.1016/j.tcb.2020.09.006 33036870

[33] Zhu L Zhao Q Yang T Ding W Zhao Y . Cellular metabolism and macrophage functional polarization. Int Rev Immunol. (2015) 34:82–100. doi: 10.3109/08830185.2014.969421 25340307

[34] Wang F Zhang S Vuckovic I Jeon R Lerman A Folmes CD . Glycolytic stimulation is not a requirement for M2 macrophage differentiation. Cell Metab. (2018) 28:463–75. doi: 10.1016/j.cmet.2018.08.012 30184486 PMC6449248

[35] Viola A Munari F Sánchez-Rodríguez R Scolaro T Castegna A . The metabolic signature of macrophage responses. Front Immunol. (2019) 10:1462. doi: 10.3389/fimmu.2019.01462 31333642 PMC6618143

[36] Gao D Bing C Griffiths HR . Disrupted adipokine secretion and inflammatory responses in human adipocyte hypertrophy. Adipocyte. (2025) 14:2485927. doi: 10.1080/21623945.2025.2485927 40176539 PMC11980453

[37] Engin A . Adipose tissue hypoxia in obesity and its impact on preadipocytes and macrophages: hypoxia hypothesis. In: Obesity and Lipotoxicity. Springer (2017). p. 305–26. 10.1007/978-3-319-48382-5_13 28585205

[38] Batista-Gonzalez A Vidal R Criollo A Carreño LJ . New insights on the role of lipid metabolism in the metabolic reprogramming of macrophages. Front Immunol. (2020) 10:2993. doi: 10.3389/fimmu.2019.02993 31998297 PMC6966486

[39] Rocha D Caldas A Oliveira L Bressan J Hermsdorff H . Saturated fatty acids trigger TLR4-mediated inflammatory response. Atherosclerosis. (2016) 244:211–5. doi: 10.1016/j.atherosclerosis.2015.11.015 26687466

[40] Rogero MM Calder PC . Obesity, inflammation, toll-like receptor 4 and fatty acids. Nutrients. (2018) 10:432. doi: 10.3390/nu10040432 29601492 PMC5946217

[41] Lumeng CN Bodzin JL Saltiel AR . Obesity induces a phenotypic switch in adipose tissue macrophage polarization. J Clin Invest. (2007) 117:175–84. doi: 10.1172/jci29881 17200717 PMC1716210

[42] Guria S Hoory A Das S Chattopadhyay D Mukherjee S . Adipose tissue macrophages and their role in obesity-associated insulin resistance: an overview of the complex dynamics at play. Biosci Rep. (2023) 43:BSR20220200. doi: 10.1042/bsr20220200 36718668 PMC10011338

[43] Nance SA Muir L Lumeng C . Adipose tissue macrophages: regulators of adipose tissue immunometabolism during obesity. Mol Metab. (2022) 66:101642. doi: 10.1016/j.molmet.2022.101642 36402403 PMC9703629

[44] Soedono S Julietta V Nawaz H Cho KW . Dynamic roles and expanding diversity of adipose tissue macrophages in obesity. J Obes Metab Syndrome. (2024) 33:193. doi: 10.7570/jomes24030 39324219 PMC11443328

[45] Jaitin DA Adlung L Thaiss CA Weiner A Li B Descamps H . Lipid-associated macrophages control metabolic homeostasis in a Trem2-dependent manner. Cell. (2019) 178:686–98. doi: 10.1016/j.cell.2019.05.054 31257031 PMC7068689

[46] Li X Ren Y Chang K Wu W Griffiths HR Lu S . Adipose tissue macrophages as potential targets for obesity and metabolic diseases. Front Immunol. (2023) 14:1153915. doi: 10.3389/fimmu.2023.1153915 37153549 PMC10154623

[47] Yu L Li Y Du C Zhao W Zhang H Yang Y . Pattern recognition receptor‐mediated chronic inflammation in the development and progression of obesity‐related metabolic diseases. Mediators Inflammation. (2019) 2019:5271295. doi: 10.1155/2019/5271295 31582899 PMC6754942

[48] Pearce EL Poffenberger MC Chang CH Jones RG . Fueling immunity: insights into metabolism and lymphocyte function. Science. (2013) 342:1242454. doi: 10.1126/science.1242454 24115444 PMC4486656

[49] Kelly B O'neill LA . Metabolic reprogramming in macrophages and dendritic cells in innate immunity. Cell Res. (2015) 25:771–84. doi: 10.1038/cr.2015.68 26045163 PMC4493277

[50] Cui C Zhang D Sun K Zhu Y Xu J Kang Y . Propofol maintains Th17/Treg cell balance in elderly patients undergoing lung cancer surgery through GABAA receptor. BMC Immunol. (2022) 23:58. doi: 10.1186/s12865-022-00490-8 36434505 PMC9701037

[51] Shan J Jin H Xu Y . T cell metabolism: a new perspective on Th17/Treg cell imbalance in systemic lupus erythematosus. Front Immunol. (2020) 11:1027. doi: 10.3389/fimmu.2020.01027 32528480 PMC7257669

[52] Bertolini TB Biswas M Terhorst C Daniell H Herzog RW Piñeros AR . Role of orally induced regulatory T cells in immunotherapy and tolerance. Cell Immunol. (2021) 359:104251. doi: 10.1016/j.cellimm.2020.104251 33248367 PMC8919860

[53] Honda K Littman DR . The microbiota in adaptive immune homeostasis and disease. Nature. (2016) 535:75–84. doi: 10.1038/nature18848 27383982

[54] Gerriets VA Kishton RJ Nichols AG Macintyre AN Inoue M Ilkayeva O . Metabolic programming and PDHK1 control CD4+ T cell subsets and inflammation. J Clin Invest. (2015) 125:194–207. doi: 10.1172/jci76012 25437876 PMC4382238

[55] Lou W Gong C Ye Z Hu Y Zhu M Fang Z . Lipid metabolic features of T cells in the tumor microenvironment. Lipids Health Dis. (2022) 21:94. doi: 10.1186/s12944-022-01705-y 36203151 PMC9535888

[56] Raud B McGuire PJ Jones RG Sparwasser T Berod L . Fatty acid metabolism in CD 8+ T cell memory: challenging current concepts. Immunol Rev. (2018) 283:213–31. doi: 10.1111/imr.12655 29664569 PMC6691976

[57] Huang H Long L Zhou P Chapman NM Chi H . mTOR signaling at the crossroads of environmental signals and T‐cell fate decisions. Immunol Rev. (2020) 295:15–38. doi: 10.1111/imr.12845 32212344 PMC8101438

[58] Hu T Liu CH Lei M Zeng Q Li L Tang H . Metabolic regulation of the immune system in health and diseases: mechanisms and interventions. Signal Transduction Targeted Ther. (2024) 9:268. doi: 10.1038/s41392-024-01954-6 39379377 PMC11461632

[59] Scaletti C Pratesi S Bellando Randone S Di Pietro L Campochiaro C Annunziato F . The B-cells paradigm in systemic sclerosis: an update on pathophysiology and B-cell-targeted therapies. Clin Exp Immunol. (2025) 219:uxae098. doi: 10.1093/cei/uxae098 39498828 PMC11754866

[60] Kumar S Dikshit M . Metabolic insight of neutrophils in health and disease. Front Immunol. (2019) 10:2099. doi: 10.3389/fimmu.2019.02099 31616403 PMC6764236

[61] de Lima Thomaz L Peron G Oliveira J da Rosa LC Thomé R Verinaud L . The impact of metabolic reprogramming on dendritic cell function. Int Immunopharmacol. (2018) 63:84–93. doi: 10.1016/j.intimp.2018.07.031 30075432

[62] Poznanski SM Barra NG Ashkar AA Schertzer JD . Immunometabolism of T cells and NK cells: metabolic control of effector and regulatory function. Inflammation Res. (2018) 67:813–28. doi: 10.1007/s00011-018-1174-3 30066126

[63] Rasaei N Heidari M Esmaeili F Khosravi S Baeeri M Tabatabaei-Malazy O . The effects of prebiotic, probiotic or synbiotic supplementation on overweight/obesity indicators: an umbrella review of the trials’ meta-analyses. Front Endocrinol. (2024) 15:1277921. doi: 10.3389/fendo.2024.1277921 38572479 PMC10987746

[64] Kim J . Regulation of immune cell functions by metabolic reprogramming. J Immunol Res. (2018) 2018:8605471. doi: 10.1155/2018/8605471 29651445 PMC5831954

[65] Wisse BE . The inflammatory syndrome: the role of adipose tissue cytokines in metabolic disorders linked to obesity. J Am Soc Nephrol. (2004) 15:2792–800. doi: 10.1097/01.ASN.0000141966.69934.21 15504932

[66] Kazankov K Jørgensen SMD Thomsen KL Møller HJ Vilstrup H George J . The role of macrophages in nonalcoholic fatty liver disease and nonalcoholic steatohepatitis. Nat Rev Gastroenterol Hepatol. (2019) 16:145–59. doi: 10.1038/s41575-018-0082-x 30482910

[67] Li J Deng X Bai T Wang S Jiang Q Xu K . Resolvin D1 mitigates non-alcoholic steatohepatitis by suppressing the TLR4-MyD88‐mediated NF‐κB and MAPK pathways and activating the Nrf2 pathway in mice. Int Immunopharmacol. (2020) 88:106961. doi: 10.1016/j.intimp.2020.106961 33182038

[68] Zhao Y Yan B Wang Z Li M Zhao W . Natural polysaccharides with immunomodulatory activities. Mini-Rev Med Chem. (2020) 20:96–106. doi: 10.2174/1389557519666190913151632 31518223

[69] Wei S Lv H Li X Xue J . Natural polysaccharides as multifunctional regulators of energy metabolism: mechanisms, structure-activity relationships, and cross-disease applications. Crit Rev Food Sci Nutr. (2026) 2026:1–30. doi: 10.1080/10408398.2026.2660145 42017809

[70] Wang XF Chen X Tang Y Wu JM Qin DL Yu L . The therapeutic potential of plant polysaccharides in metabolic diseases. Pharmaceuticals. (2022) 15:1329. doi: 10.3390/ph15111329 36355500 PMC9695998

[71] Bushueva A Adeleye T Roy P . Advancing sustainable utilization of date palm, phoenix dactylifera, a multifunctional resource for agro-industrial applications. Agric Rural Stud. (2025) 3:23. doi: 10.59978/ar02030016

[72] Bushueva A Adeleye T Roy P . Socioeconomic and environmental prospects of the food industry. Agric Rural Stud. (2024) 2:12. doi: 10.59978/ar03030017

[73] Bera M Nag PK . Integrated natural resource management and sustainable livelihoods: a review of ecosystem-based approaches for sustainability and community resilience. Agric Rural Stud. (2025) 3:23. doi: 10.59978/ar03030018

[74] Navarro DM Abelilla JJ Stein HH . Structures and characteristics of carbohydrates in diets fed to pigs: a review. J Anim Sci Biotechnol. (2019) 10:39. doi: 10.1186/s40104-019-0345-6 31049199 PMC6480914

[75] Barbosa JR de Carvalho Junior RN . Polysaccharides obtained from natural edible sources and their role in modulating the immune system: biologically active potential that can be exploited against COVID-19. Trends Food Sci Technol. (2021) 108:223–35. doi: 10.1016/j.tifs.2020.12.026 33424125 PMC7781518

[76] Fan X Li K Yang M Qin X Li Z Du Y . The influence of monosaccharide composition on the bioactivity of medicinal plant polysaccharides. Int J Mol Sci. (2026) 27:3075. doi: 10.3390/ijms27073075 41977263 PMC13073622

[77] Cheong KL Liu K Chen W Zhong S Tan K . Recent progress in Porphyra haitanensis polysaccharides: extraction, purification, structural insights, and their impact on gastrointestinal health and oxidative stress management. Food Chemistry: X. (2024) 22:101414. doi: 10.1016/j.fochx.2024.101414 38711774 PMC11070828

[78] Bi SJ Fu RJ Li JJ Chen YY Tang YP . The bioactivities and potential clinical values of Angelica sinensis polysaccharides. Nat Prod Commun. (2021) 16:1934578X21997321. doi: 10.1177/1934578x21997321

[79] Yin M Zhang Y Li H . Advances in research on immunoregulation of macrophages by plant polysaccharides. Front Immunol. (2019) 10:145. doi: 10.3389/fimmu.2019.00145 30804942 PMC6370632

[80] Xu X Wang L Zhang K Zhang Y Fan G . Managing metabolic diseases: the roles and therapeutic prospects of herb-derived polysaccharides. Biomedicine Pharmacotherapy. (2023) 161:114538. doi: 10.1016/j.biopha.2023.114538 36931026

[81] Sheng K Wang C Chen B Kang M Wang M Liu K . Recent advances in polysaccharides from Lentinus edodes (Berk.): isolation, structures and bioactivities. Food Chem. (2021) 358:129883. doi: 10.1016/j.foodchem.2021.129883 33940295

[82] Xie Y Zhang B Zhang Y . Protective effects of Acanthopanax polysaccharides on cerebral ischemia–reperfusion injury and its mechanisms. Int J Biol Macromol. (2015) 72:946–50. doi: 10.1016/j.ijbiomac.2014.09.055 25451748

[83] Szelenberger R Więckowska M . Fungal polysaccharides as modulators of molecular pathways in liver health. Molecules. (2025) 30:4384. doi: 10.3390/molecules30224384 41302443 PMC12655337

[84] Li Y Wang X Ma X Liu C Wu J Sun C . Natural polysaccharides and their derivates: a promising natural adjuvant for tumor immunotherapy. Front Pharmacol. (2021) 12:621813. doi: 10.3389/fphar.2021.621813 33935714 PMC8080043

[85] Luo M Yan J Wu L Wu J Chen Z Jiang J . Probiotics alleviated nonalcoholic fatty liver disease in high‐fat diet‐fed rats via gut microbiota/FXR/FGF15 signaling pathway. J Immunol Res. (2021) 2021:2264737. doi: 10.1155/2021/2264737 34458376 PMC8387197

[86] Cronin P Hurley C Ryan A Zamora-Úbeda M Govindan A Stanton C . Yeast β-glucan supplementation lowers insulin resistance without altering microbiota composition compared with placebo in subjects with type II diabetes: a phase I exploratory study. Br J Nutr. (2024) 132:1161–72. doi: 10.1017/s0007114524002526 39439317 PMC11617109

[87] Sami N Ahmad R Fatma T . Exploring algae and cyanobacteria as a promising natural source of antiviral drug against SARS-CoV-2. Biomed J. (2021) 44:54–62. doi: 10.1016/j.bj.2020.11.014 33640332 PMC7836382

[88] Mourelle ML Gómez CP Legido JL . The potential use of marine microalgae and cyanobacteria in cosmetics and thalassotherapy. Cosmetics. (2017) 4:46. doi: 10.3390/cosmetics4040046 30654563

[89] Bilal M Iqbal HM . Marine seaweed polysaccharides-based engineered cues for the modern biomedical sector. Mar Drugs. (2019) 18:7. doi: 10.3390/md18010007 31861644 PMC7024278

[90] Riccio G Lauritano C . Microalgae with immunomodulatory activities. Mar Drugs. (2019) 18:2. doi: 10.3390/md18010002 31861368 PMC7024220

[91] Hua H Liu L Zhu T Cheng F Qian H Shen F . Healthy regulation of Tibetan Brassica rapa L. polysaccharides on alleviating hyperlipidemia: a rodent study. Food Chemistry: Mol Sci. (2023) 6:100171. doi: 10.1016/j.fochms.2023.100171 37179738 PMC10172908

[92] Choi J Kim H-J . Preparation of low molecular weight fucoidan by gamma-irradiation and its anticancer activity. Carbohydr Polym. (2013) 97:358–62. doi: 10.1016/j.carbpol.2013.05.002 23911457

[93] Wright CM Bezabhe W Fitton JH Stringer DN Bereznicki LR Peterson GM . Effect of a fucoidan extract on insulin resistance and cardiometabolic markers in obese, nondiabetic subjects: a randomized, controlled trial. J Altern Complementary Medicine: Paradigm Practice Policy Advancing Integr Health. (2019) 25:346–52. doi: 10.1089/acm.2018.0189 30312135

[94] Zhang C Pi X Li X Huo J Wang W . Edible herbal source-derived polysaccharides as potential prebiotics: composition, structure, gut microbiota regulation, and its related health effects. Food Chem. (2024) 458:140267. doi: 10.1016/j.foodchem.2024.140267 38968717

[95] Alias AHD Shafie MH . Toward sustainable obesity management: a review of extraction, properties, and structure-activity relationships of antioxidant and anti-obesity polysaccharides. Int J Biol Macromol. (2025) 321:146171. doi: 10.1016/j.ijbiomac.2025.146171 40692053

[96] Liu C Li J Meng FY Liang SX Deng R Li CK . Polysaccharides from the root of Angelica sinensis promotes hematopoiesis and thrombopoiesis through the PI3K/AKT pathway. BMC Complementary Altern Med. (2010) 10:79. doi: 10.1186/1472-6882-10-79 21176128 PMC3022894

[97] Zhao XH Qian L Yin DL Zhou Y . Hypolipidemic effect of the polysaccharides extracted from pumpkin by cellulase-assisted method on mice. Int J Biol Macromol. (2014) 64:137–8. doi: 10.1016/j.ijbiomac.2013.12.001 24325857

[98] Yang M Yin Y Wang F Zhang H Ma X Yin Y . Supplementation with Lycium barbarum polysaccharides reduce obesity in high-fat diet-fed mice by modulation of gut microbiota. Front Microbiol. (2021) 12:719967. doi: 10.3389/fmicb.2021.719967 34512598 PMC8427603

[99] Feng Y Li H Chen C Lin H Xu G Li H . Study on the hepatoprotection of Schisandra chinensis caulis polysaccharides in nonalcoholic fatty liver disease in rats based on metabolomics. Front Pharmacol. (2021) 12:727636. doi: 10.3389/fphar.2021.727636 34621168 PMC8490749

[100] Li W Yu J Zhao J Xiao X Li W Zang L . Poria cocos polysaccharides reduces high‐fat diet‐induced arteriosclerosis in ApoE-/- mice by inhibiting inflammation. Phytother Res. (2021) 35:2220–9. 10.1002/ptr.6980 33350533

[101] Wang J Zheng D Huang F Zhao A Kuang J Ren Z . Theabrownin and Poria cocos polysaccharide improve lipid metabolism via modulation of bile acid and fatty acid metabolism. Front Pharmacol. (2022) 13:875549. doi: 10.3389/fphar.2022.875549 35833020 PMC9271858

[102] Wang G Zhang Y Zhang R Pan J Qi D Wang J . The protective effects of walnut green husk polysaccharide on liver injury, vascular endothelial dysfunction and disorder of gut microbiota in high fructose-induced mice. Int J Biol Macromol. (2020) 162:92–106. doi: 10.1016/j.ijbiomac.2020.06.055 32531370

[103] Zeng S Chen Y Wei C Tan L Li C Zhang Y . Protective effects of polysaccharide from Artocarpus heterophyllus Lam. (jackfruit) pulp on non-alcoholic fatty liver disease in high-fat diet rats via PPAR and AMPK signaling pathways. J Funct Foods. (2022) 95:105195. doi: 10.1016/j.jff.2022.105195 38826717

[104] Li Q Li W Gao Q Zou Y . Hypoglycemic effect of Chinese yam (Dioscorea opposita rhizoma) polysaccharide in different structure and molecular weight. J Food Sci. (2017) 82:2487–94. doi: 10.1111/1750-3841.13919 28906544

[105] Lovegrove A Edwards C De Noni I Patel H El S Grassby T . Role of polysaccharides in food, digestion, and health. Crit Rev Food Sci Nutr. (2017) 57:237–53. doi: 10.1080/10408398.2014.939263 25921546 PMC5152545

[106] Zhang J Xu X Liu X Chen M Bai B Yang Y . The separation, purification, structure identification, and antioxidant activity of Elaeagnus umbellata polysaccharides. Molecules. (2023) 28:6468. doi: 10.3390/molecules28186468 37764243 PMC10534330

[107] Li S Xiong Q Lai X Li X Wan M Zhang J . Molecular modification of polysaccharides and resulting bioactivities. Compr Rev Food Sci Food Saf. (2016) 15:237–50. doi: 10.1111/1541-4337.12161 33371599

[108] Wei S Lv H Li X Xue J . From molecular structure to metabolic regulation: How natural polysaccharides combat obesity via multiple pathways. Food Sci Hum Wellness. (2026) 2026:9250927. doi: 10.26599/fshw.2026.9250927

[109] Jiang W Hu Y Zhu Z . Structural characteristics of polysaccharide from Zingiber striolatum and its effects on gut microbiota composition in obese mice. Front Nutr. (2022) 9:1012030. doi: 10.3389/fnut.2022.1012030 36386925 PMC9643871

[110] Wang B Yan L Guo S Wen L Yu M Feng L . Structural elucidation, modification, and structure-activity relationship of polysaccharides in Chinese herbs: a review. Front Nutr. (2022) 9:908175. doi: 10.3389/fnut.2022.908175 35669078 PMC9163837

[111] Chan G-F Chan WK Sze D-Y . The effects of β-glucan on human immune and cancer cells. J Hematol Oncol. (2009) 2:25. doi: 10.1186/1756-8722-2-25 19515245 PMC2704234

[112] Sletmoen M Stokke BT . Higher order structure of (1, 3)‐β‐D‐glucans and its influence on their biological activities and complexation abilities. Biopolymers: Original Res Biomolecules. (2008) 89:310–21. doi: 10.1002/bip.20920 18186085

[113] Peasura N Laohakunjit N Kerdchoechuen O Vongsawasdi P Chao LK . Assessment of biochemical and immunomodulatory activity of sulphated polysaccharides from Ulva intestinalis. Int J Biol Macromol. (2016) 91:269–77. doi: 10.1016/j.ijbiomac.2016.05.062 27212215

[114] Huang L Shen M Morris GA Xie J . Sulfated polysaccharides: Immunomodulation and signaling mechanisms. Trends Food Sci Technol. (2019) 92:1–11. doi: 10.1016/j.tifs.2019.08.008 38826717

[115] Pinho SS Alves I Gaifem J Rabinovich GA . Immune regulatory networks coordinated by glycans and glycan-binding proteins in autoimmunity and infection. Cell Mol Immunol. (2023) 20:1101–13. doi: 10.1038/s41423-023-01074-1 37582971 PMC10541879

[116] Li M Huang X Wen J Chen S Wu X Ma W . Innate immune receptors co-recognition of polysaccharides initiates multi-pathway synergistic immune response. Carbohydr Polym. (2023) 305:120533. doi: 10.1016/j.carbpol.2022.120533 36737186

[117] Wu X Xin Y Zhang H Quan L Ao Q . Biopolymer-based nanomedicine for cancer therapy: opportunities and challenges. Int J Nanomed. (2024) 19:7415–71. doi: 10.2147/ijn.s460047 39071502 PMC11278852

[118] Liang X Zhou J Wang M Wang J Song H Xu Y . Progress and prospect of polysaccharides as adjuvants in vaccine development. Virulence. (2024) 15:2435373. doi: 10.1080/21505594.2024.2435373 39601191 PMC11622597

[119] Yang J Xiong K Li T Zhang M Li Z Wen Z . Anti-inflammatory effects of natural polysaccharides: molecular mechanisms and nanotherapeutic applications. Front Immunol. (2025) 16:1723346. doi: 10.3389/fimmu.2025.1723346 41472733 PMC12745254

[120] Wei W Xiao HT Bao WR Ma DL Leung CH Han XQ . TLR-4 may mediate signaling pathways of Astragalus polysaccharide RAP induced cytokine expression of RAW264. 7 cells. J Ethnopharmacol. (2016) 179:243–52. doi: 10.1016/j.jep.2015.12.060 26743224

[121] Kawai T Akira S . TLR signaling. Cell Death Differ. (2006) 13:816–25. doi: 10.1038/sj.cdd.4401850 16410796

[122] Zhou L Liu Z Wang Z Yu S Long T Zhou X . Astragalus polysaccharides exerts immunomodulatory effects via TLR4-mediated MyD88-dependent signaling pathway *in vitro* and *in vivo*. Sci Rep. (2017) 7:44822. doi: 10.1038/srep44822 28303957 PMC5355992

[123] Brown GD Taylor PR Reid DM Willment JA Williams DL Martinez-Pomares L . Dectin-1 is a major β-glucan receptor on macrophages. J Exp Med. (2002) 196:407–12. doi: 10.1084/jem.20020470 12163569 PMC2193936

[124] Brown GD Herre J Williams DL Willment JA Marshall AS Gordon S . Dectin-1 mediates the biological effects of β-glucans. J Exp Med. (2003) 197:1119–24. doi: 10.1038/mp.a004078.01 12719478 PMC2193964

[125] Taylor PR Tsoni SV Willment JA Dennehy KM Rosas M Findon H . Dectin-1 is required for β-glucan recognition and control of fungal infection. Nat Immunol. (2007) 8:31–8. doi: 10.1007/978-3-031-10898-3_25 PMC1888731 17159984

[126] Smith AJ Graves B Child R Rice PJ Ma Z Lowman DW . Immunoregulatory activity of the natural product laminarin varies widely as a result of its physical properties. J Immunol. (2018) 200:788–99. doi: 10.4049/jimmunol.1701258 29246954 PMC5760317

[127] Goodridge HS Wolf AJ Underhill DM . β‐glucan recognition by the innate immune system. Immunol Rev. (2009) 230:38–50. doi: 10.1111/j.1600-065X.2009.00793 19594628 PMC6618291

[128] Li WJ Tang XF Shuai XX Jiang CJ Liu X Wang LF . Mannose receptor mediates the immune response to Ganoderma atrum polysaccharides in macrophages. J Agric Food Chem. (2017) 65:348–57. doi: 10.1021/acs.jafc.6b04888 27931102

[129] Zimmerman JW Lindermuth J Fish PA Palace GP Stevenson TT DeMong DE . A novel carbohydrate-glycosphingolipid interaction between a β-(1–3)-glucan immunomodulator, PGG-glucan, and lactosylceramide of human leukocytes. J Biol Chem. (1998) 273:22014–20. doi: 10.1074/jbc.273.34.22014 9705343

[130] Manne BK Getz TM Hughes CE Alshehri O Dangelmaier C Naik UP . Fucoidan is a novel platelet agonist for the C-type lectin-like receptor 2 (CLEC-2). J Biol Chem. (2013) 288:7717–26. doi: 10.1074/jbc.m112.424473 23341451 PMC3597812

[131] Bachelet L Bertholon I Lavigne D Vassy R Jandrot-Perrus M Chaubet F . Affinity of low molecular weight fucoidan for P-selectin triggers its binding to activated human platelets. Biochim Biophys Acta (BBA)-General Subj. (2009) 1790:141–6. doi: 10.1016/j.bbagen.2008.10.008 19026722

[132] Bi D Zhou R Cai N Lai Q Han Q Peng Y . Alginate enhances Toll-like receptor 4-mediated phagocytosis by murine RAW264. 7 macrophages. Int J Biol Macromol. (2017) 105:1446–54. doi: 10.1016/j.ijbiomac.2017.07.129 28739412

[133] Wang B Wu B Ma Y Liu X Tao L Jia L . Astragalus polysaccharides: structure-immunomodulation relationships, multi-target pharmacological activities, and cutting-edge applications in immune modulation. Front Immunol. (2025) 16:1714898. doi: 10.3389/fimmu.2025.1714898 41383616 PMC12689378

[134] Stothers CL Burelbach KR Owen AM Patil NK McBride MA Bohannon JK . β-glucan induces distinct and protective innate immune memory in differentiated macrophages. J Immunol. (2021) 207:2785–98. doi: 10.4049/jimmunol.2100107 34740960 PMC8612974

[135] Vetvicka V Thornton BP Ross GD . Soluble beta-glucan polysaccharide binding to the lectin site of neutrophil or natural killer cell complement receptor type 3 (CD11b/CD18) generates a primed state of the receptor capable of mediating cytotoxicity of iC3b-opsonized target cells. J Clin Invest. (1996) 98:50–61. doi: 10.1172/jci118777 8690804 PMC507400

[136] Yan J Větvička V Xia Y Coxon A Carroll MC Mayadas TN . β-Glucan, a “specific” biologic response modifier that uses antibodies to target tumors for cytotoxic recognition by leukocyte complement receptor type 3 (CD11b/CD18). J Immunol. (1999) 163:3045–52. doi: 10.4049/jimmunol.163.6.3045 10477568

[137] Ross GD . Specificity of membrane complement receptor type three (CR3) for beta-glucans. Complement. (1987) 4:61–74. doi: 10.1159/000463010 3040332

[138] Adams DS Pero SC Petro JB Nathans R Mackin WM Wakshull E . PGG‐Glucan activates NF‐κB‐like and NF‐IL‐6‐like transcription factor complexes in a murine monocytic cell line. J Leukocyte Biol. (1997) 62:865–73. doi: 10.1002/jlb.62.6.86 9400829

[139] Baker RG Hayden MS Ghosh S . NF-κB, inflammation, and metabolic disease. Cell Metab. (2011) 13:11–22. doi: 10.1016/j.cmet.2010.12.008 21195345 PMC3040418

[140] Rezatabar S Karimian A Rameshknia V Parsian H Majidinia M Kopi TA . RAS/MAPK signaling functions in oxidative stress, DNA damage response and cancer progression. J Cell Physiol. (2019) 234:14951–65. doi: 10.1002/jcp.28334 30811039

[141] Copps K White M . Regulation of insulin sensitivity by serine/threonine phosphorylation of insulin receptor substrate proteins IRS1 and IRS2. Diabetologia. (2012) 55:2565–82. doi: 10.1007/s00125-012-2644-8 22869320 PMC4011499

[142] Zhao X Hou P Xin H Zhang Y Zhou A Lai C . A glucogalactomanan polysaccharide isolated from Agaricus bisporus causes an inflammatory response via the ERK/MAPK and IκB/NFκB pathways in macrophages. Int J Biol Macromol. (2020) 151:1067–73. doi: 10.1016/j.ijbiomac.2019.10.148 31739009

[143] Huang X Liu G Guo J Su Z . The PI3K/AKT pathway in obesity and type 2 diabetes. Int J Biol Sci. (2018) 14:1483–96. doi: 10.7150/ijbs.27173 30263000 PMC6158718

[144] Wu HQ Ma ZL Zhang DX Wu P Guo YH Yang F . Sequential extraction, characterization, and analysis of pumpkin polysaccharides for their hypoglycemic activities and effects on gut microbiota in mice. Front Nutr. (2021) 8:769181. doi: 10.3389/fnut.2021.769181 34805250 PMC8596442

[145] Song M Zhu X Zhao X Feng J Sui X . Plant-derived bioactive compounds in inflammation-related cancers: mechanisms and therapeutic potential. Plants. (2026) 15:575. doi: 10.3390/plants15040575 41754282 PMC12944694

[146] Sharma BR Kanneganti T-D . NLRP3 inflammasome in cancer and metabolic diseases. Nat Immunol. (2021) 22:550–9. doi: 10.1038/s41590-021-00886-5 33707781 PMC8132572

[147] Sokolova M Sjaastad I Louwe MC Alfsnes K Aronsen JM Zhang L . NLRP3 inflammasome promotes myocardial remodeling during diet-induced obesity. Front Immunol. (2019) 10:1621. doi: 10.1016/j.atherosclerosis.2019.06.209 31379826 PMC6648799

[148] Mori MA Bezy O Kahn CR . Metabolic syndrome: is Nlrp3 inflammasome a trigger or a target of insulin resistance? Circ Res. (2011) 108:1160–2. doi: 10.1161/res.0b013e318220b57b 21566220 PMC3660550

[149] Shinge SAU Zhang D Achu Muluh T Nie Y Yu F . Mechanosensitive Piezo1 channel evoked-mechanical signals in atherosclerosis. J Inflammation Res. (2021) 14:3621–36. doi: 10.2147/jir.s319789 34349540 PMC8328000

[150] Zhang S Zhang M Li W Ma L Liu X Ding Q . Research progress of natural plant polysaccharides inhibiting inflammatory signaling pathways and regulating intestinal flora and metabolism to protect inflammatory bowel disease. Int J Biol Macromol. (2023) 253:126799. doi: 10.1016/j.ijbiomac.2023.126799 37703965

[151] Cho S Ying F Sweeney G . Sterile inflammation and the NLRP3 inflammasome in cardiometabolic disease. Biomed J. (2023) 46:100624. doi: 10.1016/j.bj.2023.100624 37336361 PMC10539878

[152] Pi Y Fang M Li Y Cai L Han R Sun W . Interactions between gut microbiota and natural bioactive polysaccharides in metabolic diseases. Nutrients. (2024) 16:2838. doi: 10.3390/nu16172838 39275156 PMC11397228

[153] Yang Z Huang S Zou D Dong D He X Liu N . Metabolic shifts and structural changes in the gut microbiota upon branched-chain amino acid supplementation in middle-aged mice. Amino Acids. (2016) 48:2731–45. doi: 10.1007/s00726-016-2308-y 27539648

[154] Priyadarshini M Kotlo KU Dudeja PK Layden BT . Role of short chain fatty acid receptors in intestinal physiology and pathophysiology. Compr Physiol. (2018) 8:1091–115. doi: 10.1002/cphy.c170050 29978895 PMC6058973

[155] Morrison DJ Preston T . Formation of short chain fatty acids by the gut microbiota and their impact on human metabolism. Gut Microbes. (2016) 7:189–200. doi: 10.1080/19490976.2015.1134082 26963409 PMC4939913

[156] Yao Y Cai X Fei W Ye Y Zhao M Zheng C . The role of short-chain fatty acids in immunity, inflammation and metabolism. Crit Rev Food Sci Nutr. (2022) 62:1–12. doi: 10.1080/10408398.2020.1854675 33261516

[157] Lun W Yan Q Guo X Zhou M Bai Y He J . Mechanism of action of the bile acid receptor TGR5 in obesity. Acta Pharm Sin B. (2024) 14:468–91. doi: 10.1016/j.apsb.2023.11.011 38322325 PMC10840437

[158] Zheng X Chen T Jiang R Zhao A Wu Q Kuang J . Hyocholic acid species improve glucose homeostasis through a distinct TGR5 and FXR signaling mechanism. Cell Metab. (2021) 33:791–803:e797. doi: 10.2139/ssrn.3528695 33338411

[159] Kuang J Wang J Li Y Li M Zhao M Ge K . Hyodeoxycholic acid alleviates non-alcoholic fatty liver disease through modulating the gut-liver axis. Cell Metab. (2023) 35:1752–66:e1758. doi: 10.1016/j.cmet.2023.07.011 37591244

[160] Xiao J Dong LW Liu S Meng FH Xie C Lu XY . Bile acids-mediated intracellular cholesterol transport promotes intestinal cholesterol absorption and NPC1L1 recycling. Nat Commun. (2023) 14:6469. doi: 10.1038/s41467-023-42179-5 37833289 PMC10575946

[161] Zhang S Zhou J Wu W Zhu Y Liu X . The role of bile acids in cardiovascular diseases: from mechanisms to clinical implications. Aging Dis. (2023) 14:261. doi: 10.14336/ad.2022.0817 37008052 PMC10017164

[162] Ni Y Qian L Siliceo SL Long X Nychas E Liu Y . Resistant starch decreases intrahepatic triglycerides in patients with NAFLD via gut microbiome alterations. Cell Metab. (2023) 35:1530–47:e1538. doi: 10.1016/j.cmet.2023.08.002 37673036

[163] Alfaris N Alqahtani AM Alamuddin N Rigas G . Global impact of obesity. Gastroenterol Clinics. (2023) 52:277–93. doi: 10.1016/j.gtc.2023.03.002 37197873

[164] Ye Y Li M Chen W Wang H He X Liu N . Natural polysaccharides as promising reno-protective agents for the treatment of various kidney injury. Pharmacol Res. (2024) 207:107301. doi: 10.1016/j.phrs.2024.107301 39009291

[165] Wei H Lin X Liu L Peng X . Flaxseed polysaccharide alters colonic gene expression of lipid metabolism and energy metabolism in obese rats. Foods. (2022) 11:1991. doi: 10.3390/foods11131991 35804806 PMC9265598

[166] Yang C Xu Z Deng Q Huang Q Wang X Huang F . Beneficial effects of flaxseed polysaccharides on metabolic syndrome via gut microbiota in high-fat diet fed mice. Food Res Int. (2020) 131:108994. doi: 10.1016/j.foodres.2020.108994 32247451

[167] Hu YL Ma Q Dong X Kong Y Cai J Li J . Research progress on the therapeutic effects of polysaccharides on non-alcoholic fatty liver diseases. Front Nutr. (2023) 10:1107551. doi: 10.3389/fnut.2023.1107551 36969821 PMC10036344

[168] Lee H-B Kim Y-S Park H-Y . Pectic polysaccharides: Targeting gut microbiota in obesity and intestinal health. Carbohydr Polym. (2022) 287:119363. doi: 10.1016/j.carbpol.2022.119363 35422307

[169] Li TT Liu YY Wan XZ Huang ZR Liu B Zhao C . Regulatory efficacy of the polyunsaturated fatty acids from microalgae Spirulina platensis on lipid metabolism and gut microbiota in high-fat diet rats. Int J Mol Sci. (2018) 19:3075. doi: 10.3390/ijms19103075 30304774 PMC6213792

[170] Wan XZ Ai C Chen YH Gao XX Zhong RT Liu B . Physicochemical characterization of a polysaccharide from green microalga Chlorella pyrenoidosa and its hypolipidemic activity via gut microbiota regulation in rats. J Agric Food Chem. (2019) 68:1186–97. doi: 10.1021/acs.jafc.9b06282 31855431

[171] Wang T Han J Dai H Sun J Ren J Wang W . Polysaccharides from Lyophyllum decastes reduce obesity by altering gut microbiota and increasing energy expenditure. Carbohydr Polym. (2022) 295:119862. doi: 10.1016/j.carbpol.2022.119862 35989006

[172] Chen M Lu B Li Y Wang Y Zheng H Zhong D . Metabolomics insights into the modulatory effects of long-term compound polysaccharide intake in high-fat diet-induced obese rats. Nutr Metab. (2018) 15:8. doi: 10.1186/s12986-018-0246-2 29410697 PMC5781284

[173] Chang CJ Lin CS Lu CC Martel J Ko YF Ojcius DM . Ganoderma lucidum reduces obesity in mice by modulating the composition of the gut microbiota. Nat Commun. (2015) 6:7489. doi: 10.1038/ncomms8489 26102296 PMC4557287

[174] Amagase H Nance DM . Lycium barbarum increases caloric expenditure and decreases waist circumference in healthy overweight men and women: pilot study. J Am Coll Nutr. (2011) 30:304–9. doi: 10.1080/07315724.2011.10719973 22081616

[175] Liu L Zhang J Cheng Y Zhu M Xiao Z Ruan G . Gut microbiota: A new target for T2DM prevention and treatment. Front Endocrinol. (2022) 13:958218. doi: 10.3389/fendo.2022.958218 36034447 PMC9402911

[176] Palano F Paneni F Sciarretta S Tocci G Volpe M . Attuali concetti sullo sviluppo dell’insufficienza cardiaca nell’ipertensione. Recenti Progressi Med. (2011) 102:461. doi: 10.1701/998.10857 22258189

[177] Litwin M Kułaga Z . Obesity, metabolic syndrome, and primary hypertension. Pediatr Nephrol. (2021) 36:825–37. doi: 10.1007/s00467-020-04579-3 32388582 PMC7910261

[178] Tanase DM Gosav EM Costea CF Ciocoiu M Lacatusu CM Maranduca MA . The intricate relationship between type 2 diabetes mellitus (T2DM), insulin resistance (IR), and nonalcoholic fatty liver disease (NAFLD). J Diabetes Res. (2020) 2020:3920196. doi: 10.1155/2020/3920196 32832560 PMC7424491

[179] Suzuki A Angulo P Lymp J St Sauver J Muto A Okada T . Chronological development of elevated aminotransferases in a nonalcoholic population. Hepatology. (2005) 41:64–71. doi: 10.1002/hep.20543 15690483

[180] Yuan D Li C Huang Q Fu X Dong H . Current advances in the anti-inflammatory effects and mechanisms of natural polysaccharides. Crit Rev Food Sci Nutr. (2023) 63:5890–910. doi: 10.1080/10408398.2022.2025535 35021901

[181] Zou P Li X Wang L She Y Xiao C Peng Y . Grifola frondosa polysaccharide ameliorates inflammation by regulating macrophage polarization of liver in type 2 diabetes mellitus rats. Mol Nutr Food Res. (2024) 68:2400392. doi: 10.1101/2024.05.21.595247 39587947

[182] Lu H Luo H Li P Yang Y Cao S Zhang Y . Inulin and Lycium barbarum polysaccharides mitigate the diabetic inflammatory response by modulating bile acid metabolism to enhance regulatory T cell (Treg) activation in a rat model of diabetes. Front Endocrinol. (2025) 16:1705635. doi: 10.3389/fendo.2025.1705635 41480362 PMC12753379

[183] Tang C Zhou R Cao K Liu J Kan J Qian C . Current progress in the hypoglycemic mechanisms of natural polysaccharides. Food Funct. (2023) 14:4490–506. doi: 10.1039/d3fo00991b 37083079

[184] Chávez-Talavera O Tailleux A Lefebvre P Staels B . Bile acid control of metabolism and inflammation in obesity, type 2 diabetes, dyslipidemia, and nonalcoholic fatty liver disease. Gastroenterology. (2017) 152:1679–94:e1673. doi: 10.1053/j.gastro.2017.01.055 28214524

[185] Larsen N Vogensen FK Van Den Berg FW Nielsen DS Andreasen AS Pedersen BK . Gut microbiota in human adults with type 2 diabetes differs from non-diabetic adults. PloS One. (2010) 5:e9085. doi: 10.1371/journal.pone.0009085 20140211 PMC2816710

[186] May KS den Hartigh LJ . Modulation of adipocyte metabolism by microbial short-chain fatty acids. Nutrients. (2021) 13:3666. doi: 10.3390/nu13103666 34684670 PMC8538331

[187] Fan Y Pedersen O . Gut microbiota in human metabolic health and disease. Nat Rev Microbiol. (2021) 19:55–71. doi: 10.1038/s41579-020-0433-9 32887946

[188] Cai H Liu F Zuo P Huang G Song Z Wang T . Practical application of antidiabetic efficacy of Lycium barbarum polysaccharide in patients with type 2 diabetes. Med Chem. (2015) 11:383–90. doi: 10.2174/1573406410666141110153858 25381995 PMC4475782

[189] Zhou B Xia H Yang L Wang S Sun G . The effect of Lycium barbarum polysaccharide on the glucose and lipid metabolism: A systematic review and meta-analysis. J Am Nutr Assoc. (2022) 41:617–25. doi: 10.1080/07315724.2021.1925996 34213407

[190] Rashid R Ahmad H Ahmed Z Rashid F Khalid N . Clinical investigation to modulate the effect of fenugreek polysaccharides on type-2 diabetes. Bioact Carbohydr Dietary Fibre. (2019) 19:100194. doi: 10.1016/j.bcdf.2019.100194 38826717

[191] Abdelmalek MF . Nonalcoholic fatty liver disease: another leap forward. Nat Rev Gastroenterol Hepatol. (2021) 18:85. doi: 10.1038/s41575-020-00406-0 33420415 PMC7791336

[192] Brunt EM Kleiner DE Wilson LA Belt P Neuschwander-Tetri BA Network NCR . Nonalcoholic fatty liver disease (NAFLD) activity score and the histopathologic diagnosis in NAFLD: distinct clinicopathologic meanings. Hepatology. (2011) 53:810–20. doi: 10.1002/hep.24127 21319198 PMC3079483

[193] Farrell GC Haczeyni F Chitturi S . Pathogenesis of NASH: how metabolic complications of overnutrition favour lipotoxicity and pro-inflammatory fatty liver disease. In: Obesity, Fatty Liver and Liver Cancer (2018). p. 19–44. 10.1007/978-981-10-8684-7_3 29956204

[194] Caligiuri A Gentilini A Marra F . Molecular pathogenesis of NASH. Int J Mol Sci. (2016) 17:1575. doi: 10.3390/ijms17091575 27657051 PMC5037841

[195] Stepanova M Rafiq N Makhlouf H Agrawal R Kaur I Younoszai Z . Predictors of all-cause mortality and liver-related mortality in patients with non-alcoholic fatty liver disease (NAFLD). Digestive Dis Sci. (2013) 58:3017–23. doi: 10.1007/s10620-013-2743-5 23775317

[196] Esler WP Bence KK . Metabolic targets in nonalcoholic fatty liver disease. Cell Mol Gastroenterol Hepatol. (2019) 8:247–67. doi: 10.1016/j.jcmgh.2019.04.007 31004828 PMC6698700

[197] Sheka AC Adeyi O Thompson J Hameed B Crawford PA Ikramuddin S . Nonalcoholic steatohepatitis: a review. Jama. (2020) 323:1175–83. doi: 10.1001/jama.2020.2298 32207804

[198] van der Heide D Weiskirchen R Bansal R . Therapeutic targeting of hepatic macrophages for the treatment of liver diseases. Front Immunol. (2019) 10:2852. doi: 10.3389/fimmu.2019.02852 31849997 PMC6901832

[199] Tsomidis I Tsakou A Voumvouraki A Kouroumalis E . The role of kupffer cells and liver macrophages in the pathogenesis of metabolic dysfunction-associated steatotic liver disease. Biomedicines. (2026) 14:151. doi: 10.3390/biomedicines14010151 41595685 PMC12838681

[200] Liu S Yang X . Intestinal flora plays a role in the progression of hepatitis-cirrhosis-liver cancer. Front Cell Infect Microbiol. (2023) 13:1140126. doi: 10.3389/fcimb.2023.1140126 36968098 PMC10034054

[201] Yue SR Tan YY Zhang L Zhang BJ Jiang FY Ji G . Gynostemma pentaphyllum polysaccharides ameliorate non-alcoholic steatohepatitis in mice associated with gut microbiota and the TLR2/NLRP3 pathway. Front Endocrinol. (2022) 13:885039. doi: 10.3389/fendo.2022.885039 35937847 PMC9352886

[202] Li S Wu Y Jiang H Zhou F Ben A Wang R . Chicory polysaccharides alleviate high-fat diet-induced non-alcoholic fatty liver disease via alteration of lipid metabolism-and inflammation-related gene expression. Food Sci Hum Wellness. (2022) 11:954–64. doi: 10.1016/j.fshw.2022.03.025 38826717

[203] Chen X Liu M Tang J Wang N Feng Y Ma H . Research progress on the therapeutic effect of polysaccharides on non-alcoholic fatty liver disease through the regulation of the gut–liver axis. Int J Mol Sci. (2022) 23:11710. doi: 10.54097/1y454332 36233011 PMC9570256

[204] Hong Y Sheng L Zhong J Tao X Zhu W Ma J . Desulfovibrio vulgaris, a potent acetic acid-producing bacterium, attenuates nonalcoholic fatty liver disease in mice. Gut Microbes. (2021) 13:1930874. doi: 10.1080/19490976.2021.1930874 34125646 PMC8205104

[205] Wang X Shi L Wang X Feng Y Wang Y . MDG-1, an Ophiopogon polysaccharide, restrains process of non-alcoholic fatty liver disease via modulating the gut-liver axis. Int J Biol Macromol. (2019) 141:1013–21. doi: 10.1016/j.ijbiomac.2019.09.007 31491513

[206] Zhang B Ren D Zhao Y Liu Y Zhai X Yang X . Artemisia sphaerocephala Krasch polysaccharide prevents hepatic steatosis in high fructose-fed mice associated with changes in the gut microbiota. Food Funct. (2019) 10:8137–48. doi: 10.1039/c9fo01890e 31746883

[207] Ye H Ma S Qiu Z Huang S Deng G Li Y . Poria cocos polysaccharides rescue pyroptosis-driven gut vascular barrier disruption in order to alleviates non-alcoholic steatohepatitis. J Ethnopharmacol. (2022) 296:115457. doi: 10.1016/j.jep.2022.115457 35753609

[208] Shan-Shan S Kai W Ke M Li B Hong-Wei L . An insoluble polysaccharide from the sclerotium of Poria cocos improves hyperglycemia, hyperlipidemia and hepatic steatosis in ob/ob mice via modulation of gut microbiota. Chin J Natural Medicines. (2019) 17:3–14. doi: 10.1016/s1875-5364(19)30003-2 30704621

[209] Zhong D Xie Z Huang B Zhu S Wang G Zhou H . Ganoderma lucidum polysaccharide peptide alleviates hepatoteatosis via modulating bile acid metabolism dependent on FXR-SHP/FGF. Cell Physiol Biochem. (2018) 49:1204–20. doi: 10.1159/000493297 30196282

[210] Xing M Liu L Qian D Fang J Qi J Song P . Clinical research of fructus lycii glucopeptide on patients with nonalcoholic fatty liver disease. Zhong Xi Yi Jie He Gan Bing Za Zhi. (2008) 18:83–5.

[211] Huang H Sun Z Xu J Wang L Zhao J Li J . Yang-Xin-Shu-Mai granule alleviates atherosclerosis by regulating macrophage polarization via the TLR9/MyD88/NF-κB signaling pathway. J Ethnopharmacol. (2024) 318:116868. doi: 10.1016/j.jep.2023.116868 37454749

[212] Liu Z Yang W Wang N Wu Y . Nanomedicine targeting macrophage immunometabolism in atherosclerosis: Mechanisms and therapeutic prospects. Organ Med. (2026) 3:27–53. doi: 10.1002/orm2.70037 41531421

[213] Park SH . Regulation of macrophage activation and differentiation in atherosclerosis. J Lipid Atheroscl. (2021) 10:251. doi: 10.12997/jla.2021.10.3.251 34621697 PMC8473962

[214] Fang F Wang E Yang H Zhao T Wang Q Zhang Z . Reprogramming mitochondrial metabolism and epigenetics of macrophages via miR-10a liposomes for atherosclerosis therapy. Nat Commun. (2025) 16:9117. doi: 10.1038/s41467-025-64201-8 41087380 PMC12521651

[215] Ma C Hua Y Yang S Zhao Y Zhang W Miao Y . Wogonin attenuates atherosclerosis via KLF11‐mediated suppression of PPARα‐YAP1‐driven glycolysis and enhancement of ABCA1/G1‐mediated cholesterol efflux. Adv Sci. (2025) 12:2500610. doi: 10.1002/advs.202500610 PMC12199419 40397286

[216] Bellini R Bonacina F Norata GD . Crosstalk between dendritic cells and T lymphocytes during atherogenesis: Focus on antigen presentation and break of tolerance. Front Cardiovasc Med. (2022) 9:934314. doi: 10.3389/fcvm.2022.934314 35966516 PMC9365967

[217] Sukhorukov VN Khotina VA Chegodaev YS Ivanova E Sobenin IA Orekhov AN . Lipid metabolism in macrophages: focus on atherosclerosis. Biomedicines. (2020) 8:262. doi: 10.3390/biomedicines8080262 32752275 PMC7459513

[218] Patil NP Le V Sligar AD Mei L Chavarria D Yang EY . Algal polysaccharides as therapeutic agents for atherosclerosis. Front Cardiovasc Med. (2018) 5:153. doi: 10.3389/fcvm.2018.00153 30417001 PMC6214344

[219] Enoki T Okuda S Kudo Y Takashima F Sagawa H Kato I . Oligosaccharides from agar inhibit pro-inflammatory mediator release by inducing heme oxygenase 1. Biosci Biotechnol Biochem. (2010) 74:766–70. doi: 10.1271/bbb.90803 20378994

[220] Apostolova E Lukova P Baldzhieva A Katsarov P Nikolova M Iliev I . Immunomodulatory and anti-inflammatory effects of fucoidan: A review. Polymers. (2020) 12:2338. doi: 10.3390/polym12102338 33066186 PMC7602053

[221] Yu J Xia J Yang C Pan D Xu D Sun G . Effects of oat beta-glucan intake on lipid profiles in hypercholesterolemic adults: A systematic review and meta-analysis of randomized controlled trials. Nutrients. (2022) 14:2043. doi: 10.3390/nu14102043 35631184 PMC9147392

[222] Ho HVT Jovanovski E Zurbau A Blanco Mejia S Sievenpiper JL Au-Yeung F . A systematic review and meta-analysis of randomized controlled trials of the effect of konjac glucomannan, a viscous soluble fiber, on LDL cholesterol and the new lipid targets non-HDL cholesterol and apolipoprotein B. Am J Clin Nutr. (2017) 105:1239–47. doi: 10.3945/ajcn.116.142158 28356275

